# Neurosurgical Management of Refractory Trigeminal Neuralgia in Older Adults: A Structured Narrative Review

**DOI:** 10.3390/brainsci16101121

**Published:** 2026-10-16

**Authors:** Ahmed Najjar, Yasir Hassan Elhassan

**Affiliations:** 1 Department of General and Specialized Surgery, College of Medicine, Taibah University, Almadinah 42353, Saudi Arabia; 2 Department of Surgery, King Faisal Specialist Hospital and Research Center, Almadinah 42353, Saudi Arabia; 3 Department of Basic Medical Sciences, College of Medicine, Taibah University, Almadinah 42353, Saudi Arabia; yhassan@taibahu.edu.sa

**Keywords:** trigeminal neuralgia, older adults, microvascular decompression, balloon compression, radiofrequency rhizotomy, glycerol rhizotomy, stereotactic radiosurgery, frailty

## Abstract

**Highlights:**

**What are the main findings?**

- Observational evidence suggests that MVD may provide durable pain relief in appropriately selected, medically fit older adults.
- Percutaneous procedures and SRS are less invasive alternatives but differ in onset, durability, recurrence, and sensory complications.

**What are the implications of the main findings?**

- Chronological age alone should not automatically exclude an otherwise appropriate patient from consideration for MVD.
- Treatment should be individualized according to TN subtype, physiological reserve, comorbidities, anesthetic risk, imaging findings, pain urgency, baseline facial sensation, previous interventions, and patient preferences.

**Abstract:**

Background/Objectives: Trigeminal neuralgia (TN) disproportionately affects older adults, in whom medication intolerance, drug interactions, comorbidities, and limited physiological reserve may complicate management. This structured narrative review examines the evidence concerning the selection, effectiveness, durability, and complications of neurosurgical treatments for medication-refractory TN in older adults. Methods: PubMed was searched from database inception through 1 July 2026 using terms related to TN, older age, and neurosurgical treatment. Eligible publications evaluated microvascular decompression (MVD), radiofrequency lesioning, percutaneous balloon compression (PBC), glycerol rhizotomy, or stereotactic radiosurgery (SRS). Titles and abstracts were screened using predefined eligibility criteria, followed by further assessment of potentially eligible publications and standardized data extraction. Studies reporting separately extractable outcomes for defined older populations were classified as direct evidence; mixed-age studies without age-stratified outcomes were treated as indirect evidence and used primarily for procedural and comparative context. Results: The principal search identified 1324 unique records, and 22 publications were included: six provided direct older-adult evidence and 16 provided indirect contextual evidence. Direct evidence was largely limited to MVD and PBC. In selected older adults, MVD was associated with favorable and potentially durable pain outcomes, whereas PBC generally provided rapid initial pain relief. However, differences in outcome definitions, assessment times, follow-up, and patient selection precluded direct comparison. No included study provided separately extractable outcomes for adults aged ≥65 years undergoing radiofrequency lesioning, glycerol rhizotomy, or SRS. GRADE certainty was low or very low for most procedure-specific and comparative conclusions. Conclusions: Favorable observational outcomes in selected older patients suggest that chronological age alone should not automatically exclude an otherwise appropriate patient from consideration for MVD. Treatment should be individualized according to TN subtype, physiological reserve, comorbidity and anesthetic risk, imaging findings, pain urgency, previous interventions, baseline facial sensation, and patient preferences.

## 1. Introduction

Trigeminal neuralgia (TN) is characterized by recurrent, brief, severe electric shock-like unilateral facial pain triggered by innocuous stimuli [1]. It can substantially impair nutrition, personal care, psychological well-being, and quality of life [2]. Its incidence increases with age [3], and pain most commonly involves the maxillary and mandibular divisions [4,5]. Diagnosis is primarily clinical according to the International Classification of Headache Disorders, third edition [6]; however, bilateral symptoms, prominent sensory deficits, continuous pain, or absent triggers should prompt evaluation for alternative or secondary causes [7].

TN is classified as classical, secondary, or idiopathic. Classical TN is associated with neurovascular compression accompanied by morphological changes in the trigeminal nerve [8], whereas secondary TN results from another neurological disorder and idiopathic TN has no identifiable cause [9]. High-resolution magnetic resonance imaging is important for identifying structural lesions and clinically relevant neurovascular compression and for informing procedural selection [10]. Proposed mechanisms include focal demyelination, abnormal impulse generation, ephaptic transmission, and altered sodium-channel expression [11,12].

Carbamazepine and oxcarbazepine remain first-line treatments, but their use in older adults may be limited by adverse effects, drug interactions, and increased fall risk [13,14,15]. In this review, medication-refractory TN encompasses both persistent clinically significant pain despite an adequate trial of first-line treatment and inability to continue otherwise appropriate treatment because of adverse effects, clinically important drug interactions, or contraindications; these circumstances are clinically distinct and particularly relevant in older adults. When medication is ineffective or poorly tolerated, available interventions include microvascular decompression (MVD), radiofrequency lesioning, percutaneous balloon compression (PBC), percutaneous retrogasserian glycerol rhizotomy (PRGR), and stereotactic radiosurgery (SRS) [16,17]. These procedures differ in invasiveness, anesthetic requirements, onset and durability of pain relief, recurrence, and sensory complications.

The principal knowledge gap is the limited evidence directly applicable to older adults. Many studies involve mixed-age populations without age-stratified outcomes, apply inconsistent age thresholds and outcome definitions, and rarely assess frailty or functional status. Observational comparisons are also vulnerable to confounding by indication because medically fit older patients are more likely to undergo MVD, whereas those with greater comorbidity or operative risk are more often selected for percutaneous procedures or SRS [18,19,20,21]. Consequently, the comparative effectiveness and safety of these interventions in older adults remain uncertain.

Unlike existing guidelines, procedure-specific reviews, and meta-analyses, this review compares the principal neurosurgical modalities within a single older-adult clinical framework, explicitly separates direct older-adult evidence from indirect mixed-age evidence, and integrates evidence applicability with practical considerations relevant to individualized clinical decision-making. Accordingly, the primary review question was: among older adults aged ≥65 years with medication-refractory TN, what evidence is available concerning the selection, effectiveness, durability, medication requirements, recurrence, sensory complications, major morbidity, and mortality associated with MVD, radiofrequency lesioning or thermocoagulation, PBC, PRGR, and SRS? A secondary objective was to determine how indirect evidence from mixed-age populations could inform procedural context and individualized clinical decision-making when direct older-adult evidence was unavailable. This structured narrative review critically examines the available evidence and proposes an individualized framework for treatment discussion.

## 2. Materials and Methods

### 2.1. Review Design

This study was conducted as a structured narrative review of neurosurgical treatments for medication-refractory trigeminal neuralgia in older adults. Structured search, eligibility, selection, extraction, and methodological-appraisal procedures were used to improve transparency and reproducibility. Nevertheless, the review was designed for critical interpretive synthesis rather than as a formal systematic review. The protocol was not prospectively registered, PubMed/MEDLINE was the sole bibliographic database searched, indirect contextual evidence was included according to predefined criteria, and no quantitative meta-analysis was applied. A narrative approach was retained because of substantial heterogeneity in age definitions, definitions of medication refractoriness, TN classification, patient selection, procedural techniques, outcome measures, and follow-up duration.

### 2.2. Information Sources and Search Strategy

A structured literature search was conducted in PubMed/MEDLINE from database inception to 1 July 2026. PubMed/MEDLINE was the sole bibliographic database searched. Although this provided a reproducible and auditable search dataset, the absence of searches in other major databases, such as Embase, Scopus, or Web of Science, may have resulted in the omission of relevant publications. Accordingly, the search should not be regarded as a comprehensive search of all available literature.

The search combined controlled vocabulary, where available, with free-text terms relating to TN, older age, and neurosurgical treatment. The principal search strategy was:

(“trigeminal neuralgia” OR “tic douloureux”) AND (“older adult*” OR elder* OR aged OR geriatric* OR octogenarian*) AND (“microvascular decompression” OR MVD OR “radiofrequency rhizotomy” OR “radiofrequency lesion*” OR “radiofrequency thermocoagulation” OR “percutaneous balloon compression” OR “balloon compression” OR “glycerol rhizotomy” OR “glycerol rhizolysis” OR “stereotactic radiosurgery” OR “Gamma Knife” OR “CyberKnife” OR neurosurgery*)

The complete PubMed search syntax, date of execution, and number of retrieved records are reported in Supplementary Table S1. Reference lists of eligible studies, relevant clinical guidelines, systematic reviews, and meta-analyses were also examined to identify additional publications.

A supplementary search that omitted age-related terms retrieved 3239 records and was maintained as a separate search stream because it served a different purpose from the principal age-focused search. The principal search was designed to identify evidence directly relevant to older adults, whereas the broader supplementary search was designed to identify mixed-age evidence providing procedural and comparative context when direct older-adult evidence was unavailable. Maintaining separate streams preserved the provenance and directness of the evidence and prevented contextual studies from being interpreted as age-specific evidence. The supplementary records were deduplicated by PMID against the 1324 principal-search records; 1317 overlapped, leaving 1922 unique records. All 1922 records were independently screened by two reviewers using predefined eligibility criteria. Eligible contextual sources were restricted to evidence-based clinical guidelines, systematic reviews or meta-analyses evaluating the included procedures, direct comparative studies of eligible interventions, and primary studies reporting quantitative data for a prespecified procedure–outcome combination for which no eligible direct older-adult publication reported quantitative data. The prespecified domains were onset of pain relief, durability, recurrence, medication requirements, sensory complications, major morbidity, and mortality. Selection was not based on perceived influence, citation frequency, or direction of findings, and disagreements were resolved through discussion and consensus. The supplementary search contributed five publications not identified by the principal search. These publications were used only as indirect contextual evidence and did not contribute to older-adult-specific outcome estimates.

Eligibility was restricted to articles published in English; non-English-language publications were not assessed for inclusion. No publication-date restriction was applied. Articles published electronically or in print by 1 July 2026 were eligible for consideration.

### 2.3. Eligibility Criteria

Studies were considered eligible when they:

1. Included adults with classical, idiopathic, or secondary TN;
2. Evaluated microvascular decompression (MVD), radiofrequency lesioning or thermocoagulation, percutaneous balloon compression (PBC), percutaneous retrogasserian glycerol rhizotomy (PRGR), or stereotactic radiosurgery (SRS);
3. Either (a) included a population defined by the original investigators as older adults or reported separately extractable age-stratified outcomes, or (b) met the predefined criteria for indirect contextual evidence by being an evidence-based clinical guideline, a systematic review or meta-analysis of an eligible procedure, a direct comparative study of eligible interventions, or a primary study reporting a prespecified procedure–outcome combination for which no eligible direct older-adult publication provided quantitative data;
4. Reported at least one clinically relevant outcome, including pain freedom, pain reduction, medication use, recurrence, treatment durability, facial sensory disturbance, cranial nerve dysfunction, major morbidity, or mortality;
5. Were published as clinical trials, prospective or retrospective cohort studies, comparative studies, case series, systematic reviews, meta-analyses, or evidence-based clinical guidelines.

For this review, older adults were operationally defined as individuals aged ≥65 years. Studies restricted to participants aged ≥65 years or reporting separately extractable outcomes for this age group were classified as direct older-adult evidence. Studies applying higher thresholds, including ≥70 or ≥75 years, were retained, but their exact age definitions were recorded, and their findings were reported separately. Studies using a threshold below 65 years, elderly-dominant cohorts without separately extractable outcomes for participants aged ≥65 years, and general mixed-age studies were classified as indirect evidence. A high mean or median age without an explicit age threshold or separately extractable age-stratified outcomes was insufficient for classification as direct older-adult evidence.

General-population studies identified through the supplementary search were classified as indirect contextual evidence. Such studies were eligible only when they were evidence-based clinical guidelines, systematic reviews or meta-analyses of eligible procedures, direct comparative studies of included interventions, or studies addressing a prespecified procedural outcome that was not adequately reported in direct older-adult studies. The prespecified outcome domains were onset of pain relief, durability, recurrence, medication requirements, sensory complications, major morbidity, and mortality. Indirect evidence was used only to describe procedural characteristics or provide comparative context; it was not used to calculate older-adult outcome estimates or infer age-specific treatment effects.

Studies were excluded if they:

1. Exclusively examined pediatric populations;
2. Addressed facial pain disorders other than TN without reporting TN-specific findings;
3. Evaluated medical or complementary treatments without relevant neurosurgical outcomes;
4. Reported experimental or obsolete procedures with no current clinical relevance;
5. Lacked sufficient information regarding the intervention or clinical outcomes;
6. Were conference abstracts, editorials, letters without original data, or isolated case reports; or
7. Were not available in English.

For this review, medication-refractory TN was operationally defined as persistent clinically significant pain despite an adequate trial of carbamazepine or oxcarbazepine at the maximum tolerated dose, or inability to continue an otherwise appropriate first-line treatment because of treatment-limiting adverse effects, clinically important drug interactions, or contraindications. An adequate trial was interpreted according to the dose and duration reported by each study because no uniform regimen was used across the literature. Studies requiring failure of additional medications were retained, and their definitions were recorded separately. Refractoriness was categorized as inadequate efficacy, medication intolerance or contraindication, both, or insufficiently specified. These categories were not considered clinically interchangeable.

### 2.4. Study Selection

After duplicate checking, two reviewers independently screened the titles and abstracts of the 1324 records retrieved through the principal search. During this preliminary screening, 331 records were excluded, 253 were classified as potentially eligible, and 740 were classified as uncertain because eligibility could not be determined conclusively from the title and abstract. Thus, 993 records were retained for further eligibility assessment; this figure does not represent 993 confirmed full-text articles. Full texts were obtained when required to determine eligibility; however, the exact number of full-text articles retrieved and assessed was not prospectively recorded and could not be reconstructed reliably.

The 1922 records unique to the supplementary search were screened separately by the same two reviewers using the predefined eligibility criteria for indirect contextual evidence. Full texts were obtained when required to determine eligibility, and potentially eligible publications were independently assessed by both reviewers. Disagreements were resolved through discussion and consensus. Seventeen publications identified through the principal-search stream and five uniquely identified through the supplementary-search stream were ultimately included.

**Figure 1:**
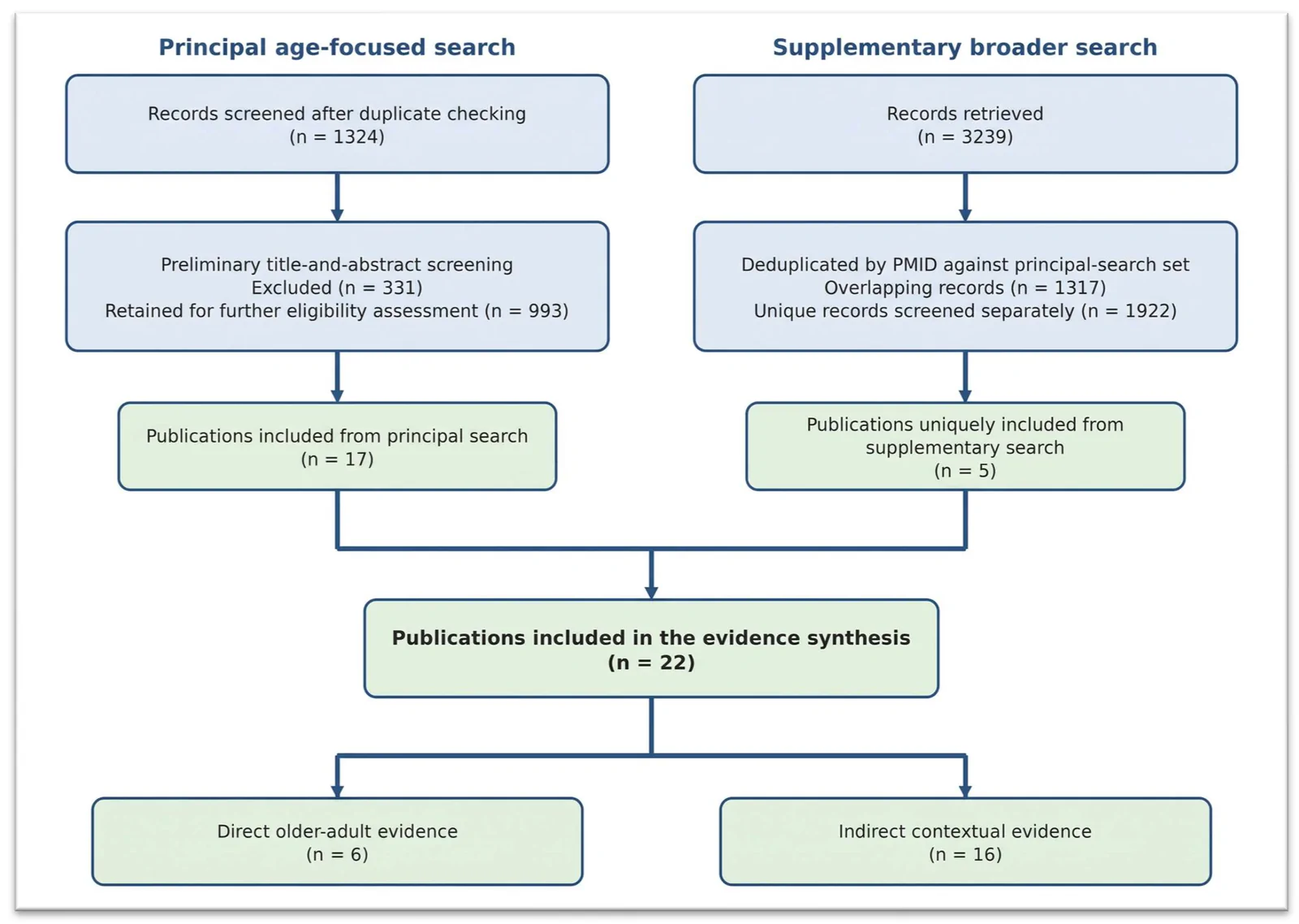
Study-selection flow diagram for the principal age-focused PubMed search and supplementary broader search. The principal search retrieved 1324 records, of which 331 were excluded during preliminary title-and-abstract screening, and 993 were retained for further eligibility assessment. These 993 records did not represent 993 confirmed full-text articles. Full texts were obtained when required, but the exact number retrieved and assessed was not prospectively recorded and could not be reconstructed reliably. The supplementary search retrieved 3239 records; 1317 overlapped with the principal-search set, leaving 1922 unique records that were screened separately. Seventeen publications were included from the principal search, and five were uniquely included from the supplementary search, resulting in 22 publications in the evidence synthesis: six providing direct older-adult evidence and 16 providing indirect contextual evidence. Detailed record-level reasons for exclusion during further eligibility assessment were not prospectively documented and could not be reconstructed reliably. Because detailed record-level reasons for exclusion during further eligibility assessment were not prospectively documented, the flow diagram does not provide the level of exclusion tracking normally expected from a formal systematic review.

Detailed record-level reasons for exclusion during further eligibility assessment were not prospectively documented and could not be reconstructed reliably; therefore, retrospective exclusion categories were not assigned. Consequently, the study-selection flow does not provide the level of record-level exclusion tracking normally expected from a formal systematic review. Figure 1 reports the numbers of records identified, the overlap between the two search streams, the preliminary screening decisions, the records retained for further eligibility assessment, and the publications ultimately included.

When multiple publications appeared to describe overlapping patient populations, we prioritized the most comprehensive or recent report. Earlier reports were retained only when they provided additional outcomes or longer follow-up.

Records designated for further assessment during title-and-abstract screening were not considered included studies. Only publications that subsequently satisfied the full eligibility criteria and were retained in the evidence synthesis were counted as included studies.

### 2.5. Data Collection

Relevant information was collected using a standardized data-extraction framework. The following variables were recorded when available:

- Study design and publication year;
- Sample size and study setting;
- Definition of older age;
- Age threshold or age-stratification method supporting that classification;
- Reason for including an indirect source and the prespecified outcome domain it addressed;
- Chronological age, comorbidity burden, reported anesthetic-risk classification, functional status, and frailty assessment, including the instrument used when available;
- TN classification and affected trigeminal divisions;
- Previous medical or procedural treatments;
- Definition of medication-refractory TN, including medications attempted, dose and duration when reported, inadequate efficacy, intolerance, contraindications, and reasons for treatment discontinuation;
- Intervention and relevant technical characteristics;
- Duration of follow-up;
- Definition and timing of pain relief;
- Medication-free pain freedom or continued medication use;
- Recurrence rate and time to recurrence;
- Facial numbness, dysesthesia, corneal sensory impairment, and anesthesia dolorosa;
- Cranial nerve deficits and procedure-specific complications;
- Major morbidity and mortality; and
- Methodological limitations relevant to interpretation.

When a study did not report the medications attempted or the criteria used to establish treatment failure or intolerance, its definition of medication refractoriness was classified as insufficiently specified.

One reviewer performed the initial data extraction using the standardized framework. The second reviewer independently checked the extracted study characteristics, age classifications, definitions of medication refractoriness, reported outcomes, classification as direct older-adult or indirect contextual evidence, and the stated reason for including each indirect source. Disagreements were resolved through discussion, consensus, and consultation of the original publication.

Pain outcomes were preferentially reported using the Barrow Neurological Institute pain intensity scale when available: BNI I, no pain without medication; BNI II, occasional pain without medication; BNI III, some pain adequately controlled with medication; and BNI IV–V, inadequately controlled or no pain relief. Outcomes reported using other definitions were retained in the original authors’ terminology and were not converted to BNI categories unless an unambiguous mapping was possible. Immediate outcomes and outcomes at specified or mean follow-up intervals were reported separately. Terms such as “success,” “efficacy,” “meaningful pain relief,” “excellent outcome,” and “favorable outcome” were treated as author-defined outcomes and were accompanied by the original definition and assessment time whenever these were reported; they were not interpreted as BNI I or complete medication-free pain freedom unless the source explicitly supported that interpretation.

The completed study-level extraction table for all 22 included publications is provided in Supplementary Table S2. For each publication, it reports the number of participants, age or age threshold, follow-up duration, study design, intervention, outcome definitions, principal findings, and classification as direct older-adult or indirect contextual evidence. Where a specific data point was unavailable in the original source, it is identified as “not reported”.

### 2.6. Methodological Quality and Risk-of-Bias Assessment

Methodological quality and risk of bias were assessed using design-appropriate instruments. Randomized studies were evaluated using the Cochrane Risk of Bias 2 (RoB 2) tool. Nonrandomized comparative intervention studies and observational cohorts estimating intervention effects were evaluated using the Risk Of Bias In Non-randomized Studies—of Interventions (ROBINS-I) tool. Uncontrolled descriptive case series were evaluated using the Joanna Briggs Institute Critical Appraisal Checklist for Case Series because ROBINS-I is not specifically designed for purely descriptive studies without a comparator. For case series, the appraisal considered clear eligibility criteria, reliable and consistent measurement of the condition and outcomes, consecutive and complete inclusion of participants, adequate reporting of clinical and demographic characteristics and outcomes, and appropriate statistical analysis. The JBI checklist findings were reported using item-level judgments and supporting rationales and were not converted into ROBINS-I overall risk-of-bias categories. Systematic reviews and meta-analyses were assessed using AMSTAR 2. Publications containing both a primary observational study and a systematic review or meta-analysis were assessed using the instruments applicable to each component. Study-level judgments, the instrument applied to each publication, and supporting rationales are presented in Supplementary Table S3. Risk-of-bias and methodological-quality findings informed the narrative synthesis and interpretation of the conclusions rather than serving as criteria for study exclusion.

The certainty of evidence was evaluated using the GRADE domains of risk of bias, inconsistency, indirectness, imprecision, and publication bias. Evidence bodies were defined according to the procedure, population and evidence directness, and clinically relevant outcome domain. The critical outcomes were pain relief, onset of pain relief, durability or recurrence, medication requirements, facial sensory complications, major morbidity, and mortality. Comparative effectiveness and the reduction in the trigeminocardiac reflex with trigeminal ganglion blockade during PBC were assessed as separate evidence bodies because they addressed distinct clinical questions. Direct older-adult evidence was assessed separately from evidence derived exclusively or predominantly from mixed-age populations. Because quantitative pooling was not undertaken, certainty judgments were applied narratively to these procedure–outcome evidence bodies rather than to pooled effect estimates. Evidence derived from randomized studies initially started at high certainty, whereas evidence derived from observational studies initially started at low certainty. Systematic reviews did not automatically determine the starting certainty; the design and limitations of their underlying studies were considered. Certainty was downgraded when concerns were identified regarding risk of bias, inconsistency across studies or outcome definitions, indirectness to adults aged ≥65 years or to the specified outcome, imprecision arising from small samples or few events, and possible publication bias. No upgrading was applied because the evidence did not demonstrate a large effect resistant to plausible residual confounding, a dose–response gradient, or an effect strengthened after accounting for plausible confounding. Final certainty was categorized as high, moderate, low, or very low, with outcome-specific judgments and supporting rationales reported in Supplementary Table S3.

### 2.7. Evidence Synthesis

The evidence was organized according to treatment modality: MVD, radiofrequency lesioning or thermocoagulation, PBC, PRGR, and SRS. Procedures were compared by onset and durability of pain relief, recurrence, medication requirements, sensory complications, anesthesia requirements, procedural burden, and suitability for older adults. Direct older-adult evidence was organized according to the age threshold used by each study, including ≥65, ≥70, ≥75, or ≥80 years. Outcomes based on different age thresholds were not assumed to represent equivalent populations. Mixed-age evidence without separately extractable outcomes for participants aged ≥65 years was explicitly classified as indirect evidence. Studies defining refractoriness by inadequate efficacy, medication intolerance, or both were identified separately. Because drug regimens and definitions varied substantially, these populations were not assumed to be equivalent, and no pooled estimate based on refractory status was calculated.

Direct older-adult evidence and indirect contextual evidence were synthesized separately. Conclusions concerning treatment selection and outcomes in older adults were based primarily on direct evidence. Indirect evidence was used only to describe general procedural characteristics or to provide context for prespecified outcomes when direct older-adult evidence was unavailable. It was not interpreted as demonstrating equivalent effectiveness or safety in older adults.

To avoid double counting, primary studies, systematic reviews or meta-analyses, and clinical guidelines were not treated as independent observations of equivalent evidentiary weight. When an included primary study also contributed to an included systematic review or meta-analysis, study-specific outcomes were extracted from and attributed to the primary publication. The review-level source was used only to provide broader procedural context, evaluate consistency or heterogeneity across studies, or address a prespecified outcome not adequately covered by the included primary studies. Review-level pooled estimates were not combined with, or counted as additional confirmation of, findings from their constituent primary studies. Clinical guidelines were used only for contextual recommendations and did not contribute independent procedural outcome estimates.

A narrative synthesis was undertaken because substantial clinical and methodological heterogeneity precluded meaningful quantitative pooling. In particular, the studies differed in age thresholds, TN subtype, previous treatments, procedural techniques, definitions of pain relief, follow-up duration, and reporting of complications. Greater interpretive weight was given to evidence directly reporting outcomes for predefined older populations, followed by comparative studies, larger cohorts, systematic reviews, meta-analyses, and clinical guidelines. This qualitative weighting was informed by the formal risk-of-bias and GRADE certainty assessments reported in Supplementary Table S3.

### 2.8. Use of Generative Artificial Intelligence

During manuscript preparation, the author used Super Genius AI Chat by DeepAI (web-based version; accessed on multiple occasions between September and October 2026).to assist with manuscript organization, language revision, and improvement of clarity and readability. The tool was not used as a source of scientific evidence or to conduct database searches, study selection, data extraction, evidence assessment, or interpretation of the findings. The author independently verified the scientific content and references, critically revised the output, and assumes full responsibility for the final manuscript.

## 3. Results

### 3.1. Characteristics of Evidence

The principal PubMed/MEDLINE search yielded 1324 unique records. Of these, 331 were excluded during preliminary title-and-abstract screening, and 993 records were retained for further eligibility assessment. These 993 records included 253 classified as potentially eligible and 740 classified as uncertain; they did not represent 993 confirmed full-text articles. Full texts were obtained when required to determine eligibility, but the exact number retrieved and assessed was not prospectively recorded and could not be reconstructed reliably. The supplementary search retrieved 3239 records, of which 1317 overlapped with the principal-search set. The remaining 1922 unique records were screened separately using the predefined criteria for indirect contextual evidence. Of the 22 publications ultimately included in the evidence synthesis, 17 were identified by the principal search, and five were contributed uniquely by the supplementary search: Sarsam et al. [22], Yang et al. [23], Gunduz et al. [24], Wu et al. [25], and Kodeeswaran et al. [26]. By study design, these comprised one randomized controlled study, two systematic reviews and meta-analyses, one combined prospective study and systematic review/meta-analysis, two prospective comparative cohort studies, and 16 other observational cohort, comparative, or case-series publications. By procedural focus, five publications evaluated microvascular decompression (MVD), three evaluated radiofrequency lesioning or thermocoagulation, two evaluated percutaneous balloon compression (PBC), two evaluated percutaneous retrogasserian glycerol rhizotomy (PRGR), three evaluated stereotactic radiosurgery (SRS), three directly compared percutaneous procedures, and four compared MVD with SRS. These mutually exclusive categories total 22 publications. Six publications provided direct older-adult evidence, whereas 16 involved mixed-age or general adult populations and were classified as indirect contextual evidence. Of the six direct older-adult publications, four evaluated MVD and two evaluated PBC-related outcomes. Although this review used ≥65 years as its primary operational definition of an older adult, the age thresholds used by the included studies varied and commonly included ≥65, ≥70, and ≥75 years. Results were therefore reported according to each study’s exact age threshold rather than combined as though they represented a homogeneous older population. Supplementary Table S2 provides study-level information for all 22 included publications, including participant numbers, age or age thresholds, and follow-up duration. These data permit cross-verification of the procedure-level evidence summaries presented in Supplementary Table S4; unavailable source data are explicitly identified as “not reported”.

Age thresholds, definitions of medication refractoriness, outcome measures, and follow-up periods varied across the included publications; source-specific details are provided in Supplementary Table S2. None of the included publications measured frailty using a validated instrument and analyzed its association with procedural outcomes. Although some studies reported selected comorbidities or perioperative risk variables, the available data did not establish whether frailty or comorbidity predicted outcomes more accurately than chronological age.

The identified procedures were classified into three principal categories:

- Open non-ablative surgery: microvascular decompression (MVD);
- Percutaneous ablative procedures: radiofrequency lesioning or thermocoagulation, percutaneous balloon compression (PBC), and percutaneous retrogasserian glycerol rhizotomy (PRGR);
- Non-invasive ablative treatment: stereotactic radiosurgery (SRS).

Figure 1 illustrates the principal procedural approaches, and Table 1 summarizes their comparative clinical characteristics.

Supplementary Table S4 summarizes the number of publications and participants, age criteria, follow-up, evidence directness, and GRADE certainty for each procedure.

### 3.2. Methodological Quality and Risk of Bias

Overall, the evidence base was methodologically heterogeneous. The appraisal instrument was selected according to study design: ROBINS-I was applied to nonrandomized studies estimating intervention effects, whereas the JBI Critical Appraisal Checklist for Case Series was applied to uncontrolled descriptive case series. Most primary studies were retrospective, nonrandomized, and conducted at single centers, resulting in concerns regarding participant selection, uncontrolled confounding, treatment-selection bias, and incomplete or inconsistently reported follow-up. Comparative studies were particularly susceptible to confounding by indication because patients receiving microvascular decompression, percutaneous procedures, and stereotactic radiosurgery frequently differed in age, frailty, comorbidity, previous treatment, and trigeminal neuralgia phenotype. The systematic reviews provided broader evidence but were limited by the quality and heterogeneity of their included studies. Only one included randomized study was identified, and its scope was limited to a specific perioperative question rather than comparative long-term procedural effectiveness. Consequently, the certainty of evidence supporting direct comparisons and treatment-selection recommendations was generally low.

For GRADE, the principal evidence bodies were defined by procedure and critical outcome and were further distinguished according to whether they provided direct older-adult evidence or indirect mixed-age evidence. These bodies addressed MVD pain relief and durability; MVD major complications and mortality; PBC pain relief and durability; reduction in the trigeminocardiac reflex during PBC; the effectiveness, durability, and sensory complications of radiofrequency lesioning, PRGR, and SRS; and comparative effectiveness among MVD, SRS, and percutaneous procedures. The predominantly observational evidence began at low certainty. The randomized evidence concerning trigeminal ganglion blockade during PBC began at high certainty but was downgraded for methodological concerns and imprecision. MVD evidence was downgraded principally for risk of bias, heterogeneous outcomes and follow-up, and imprecision. Evidence concerning PBC was downgraded for risk of bias, indirect longer-term comparisons, and imprecision. Evidence concerning radiofrequency lesioning, PRGR, SRS, and between-procedure comparisons was downgraded for risk of bias, inconsistency, indirectness to older adults, and imprecision. Consequently, certainty was very low for most procedure-specific and comparative conclusions and low for the specific randomized perioperative PBC finding.

Study-level risk-of-bias assessments are presented in Part A of Supplementary Table S3. Part B provides the outcome-specific GRADE assessment, including reasoned judgments for risk of bias, inconsistency, indirectness, imprecision, and publication bias, together with the resulting certainty rating for each principal evidence body.

### 3.3. Microvascular Decompression

**Figure 2:**
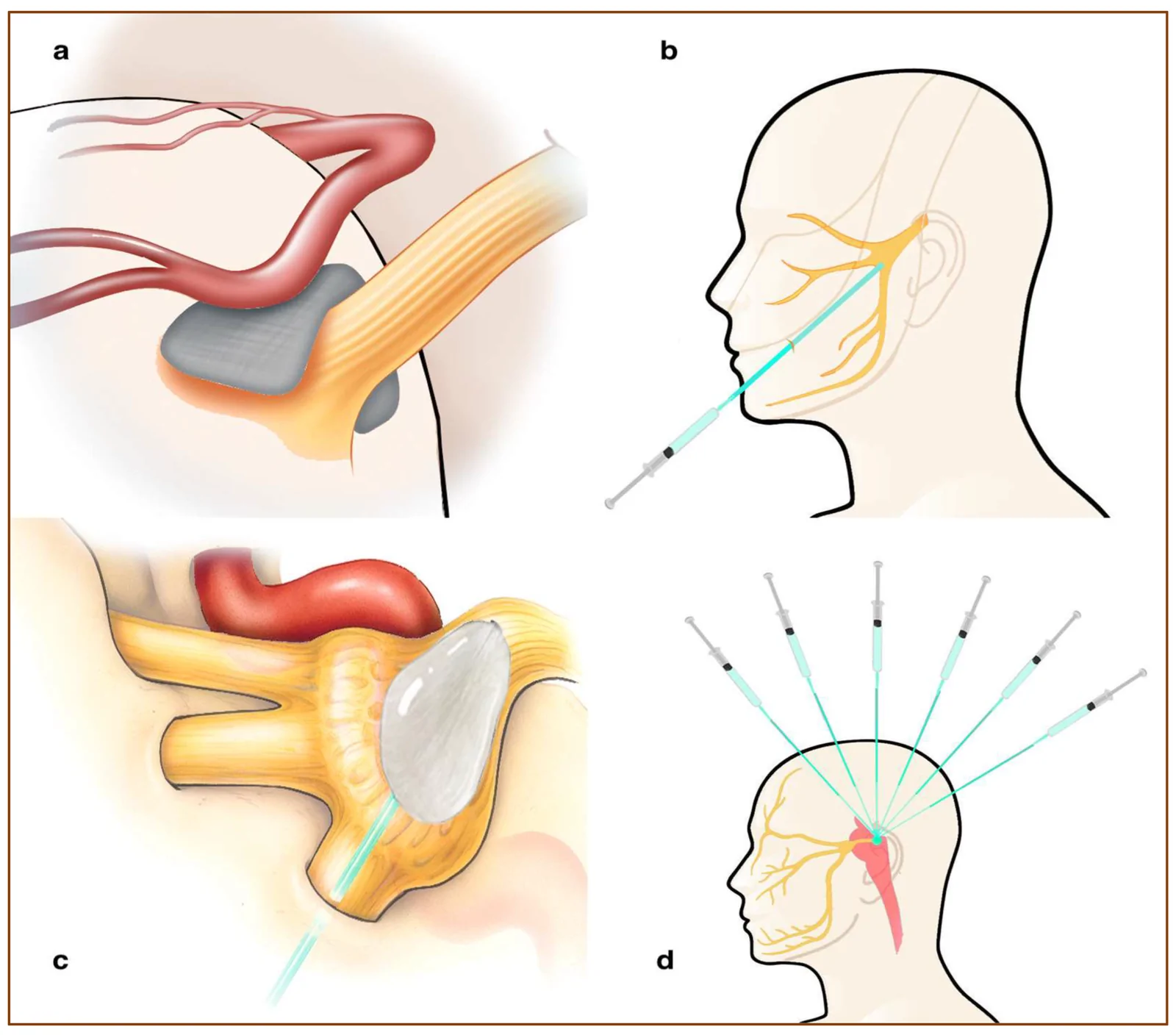
Principal invasive and non-invasive procedures used to treat refractory trigeminal neuralgia: (**a**) microvascular decompression; (**b**) percutaneous radiofrequency lesioning; (**c**) percutaneous balloon compression; and (**d**) stereotactic radiosurgery. Original illustration prepared by Dr. Leena Babiker specifically for this review and reproduced with her permission.

MVD addresses the presumed cause of classical TN by separating the compressing vessel from the trigeminal nerve, commonly through the placement of an interposition material between the two structures (Figure 2a). Unlike ablative procedures, MVD is intended to preserve trigeminal nerve function. Its effectiveness is greatest in patients with classical TN and radiological or intraoperative evidence of neurovascular compression accompanied by morphological changes in the affected nerve [8,10,16].

MVD outcomes were reported using heterogeneous definitions. Ferroli et al. reported complete pain relief without medication in 84.5% of 117 patients aged >65 years over 7–138 months of follow-up [18]; this outcome most closely corresponds to BNI I. Menna et al. reported immediate pain freedom at hospital discharge in 25/25 patients aged ≥65 years. At follow-up (15.60 ± 12.06 months; overall range, 3–48 months), the mean BNI score was 1.52 ± 1.15, and recurrence occurred in 4/25 patients (16%) [19]. Because categorical BNI results were not reported, the mean score was not converted into a medication-free pain-freedom rate. Sekula et al. reported an “excellent outcome” in 31/36 older patients (86.1%) at a mean follow-up of 20.0 ± 7.0 months [20]. This original terminology was retained because the available report did not establish that all 31 patients met BNI I or were medication-free. In the meta-analysis by Sun et al., pooled immediate and long-term efficacy rates among older patients were 92.7% and 77.5%, respectively [21]. These estimates were retained as author-defined efficacy outcomes rather than interpreted as BNI I because definitions and follow-up intervals varied among the included studies.

Observational studies have reported more durable pain control after MVD than after percutaneous procedures or SRS. However, direct age-specific comparisons are sparse, and differences in patient selection prevent these findings from establishing comparative superiority in older adults. Long-term observational evidence indicates that recurrence occurs most frequently during the first two postoperative years, with the annual recurrence rate declining during longer follow-up [22]. Less favorable outcomes have been associated with continuous or atypical pain, prolonged symptom duration, venous rather than arterial compression, and the absence of convincing morphological nerve changes.

The principal disadvantages of MVD are the need for general anesthesia, posterior fossa craniotomy, and postoperative hospitalization. In the direct older-adult cohort reported by Ferroli et al. (117 patients aged >65 years), slight trigeminal hypoesthesia occurred in 5.8%, severe hearing loss in 0.9%, cerebrospinal fluid leakage in 4.2%, transient diplopia in 3.4%, and posterior fossa subdural hematoma in 0.8%; no deaths occurred [18]. In Menna et al., among 25 patients aged ≥65 years, cerebrospinal fluid leakage occurred in 2/25 (8%) and other postoperative complications in 2/25 (8%) [19]. The study reported specific recovery times only for the overall mixed-age cohort: hemiparesis caused by pontine ischemia resolved within two months, diplopia within one week, transient hearing loss within two weeks, and hypoesthesia within three weeks; one chronic subdural hematoma required surgical drainage. In the prospective older cohort of Sekula et al. (n = 36), no cerebrospinal fluid leaks, cerebellar hematomas, strokes, or deaths were reported [20]. These estimates arose from selected older surgical candidates and should not be generalized to all older adults, particularly those with substantial frailty or cardiopulmonary comorbidity. These favorable outcomes in selected surgical cohorts do not establish that age is irrelevant before patient selection; they suggest only that chronological age should not be used as an automatic contraindication in otherwise appropriate candidates [18,19,20,21].

### 3.4. Percutaneous Ablative Procedures

Percutaneous procedures access the trigeminal ganglion through the foramen ovale and intentionally injure trigeminal fibers to interrupt pain transmission. In contrast to MVD, these procedures do not correct an underlying neurovascular conflict. Their principal advantages are rapid pain relief, shorter hospitalization, and a lower physiological burden. Their main disadvantages are facial sensory disturbance and a greater probability of recurrent pain [17].

#### 3.4.1. Radiofrequency Lesioning

Radiofrequency lesioning, also termed radiofrequency thermocoagulation, uses controlled thermal energy to produce a lesion in selected trigeminal fibers or the trigeminal ganglion (Figure 2b). Sensory stimulation before lesioning may help identify the affected trigeminal division and permit a degree of anatomical selectivity.

No included study provided separately extractable radiofrequency-lesioning outcomes for a population defined as aged ≥65 years. The available outcome data were therefore classified as indirect contextual evidence and should not be interpreted as estimates of effectiveness or safety specifically in older adults. In a mixed-age cohort, Yang et al. reported an author-defined overall success rate of approximately 80% after percutaneous radiofrequency treatment [23]. This estimate is presented only to describe the general procedural evidence and was not used to infer an older-adult treatment effect. Mixed-age studies also suggested that treatment temperature may influence effectiveness and sensory complications: higher temperatures may create more extensive lesions but increase facial numbness, dysesthesia, and other sensory injury, particularly at temperatures exceeding approximately 70 °C [24,32]. These temperature-related findings likewise constitute indirect evidence.

Quantitative complication data for radiofrequency lesioning were available only from mixed-age populations. In the series by Gunduz et al., early facial hypoesthesia occurred after 193/209 procedures (92.3%). This total comprised two mutually exclusive categories reported by the authors: tactile hypoesthesia after 182/209 procedures (87.1%) and facial numbness after 11/209 procedures (5.3%). Facial numbness was therefore a subgroup of facial hypoesthesia rather than an additional event. The source described these as differing degrees of sensory change but did not provide further operational definitions distinguishing them. Dysesthesia was reported separately after 3/209 procedures (1.4%). Corneal hypoesthesia or reflex impairment occurred after 7/209 procedures (3.3%), masseter weakness after 5/209 (2.4%), and temporal-muscle atrophy after 1/209 (0.5%). These were early procedural outcomes, and their persistence was not consistently reported. Yang et al. reported no diplopia, keratitis, or anesthesia dolorosa at long-term follow-up among 24 patients undergoing radiofrequency treatment after previous invasive treatment; however, this small subgroup was not restricted to older adults [23]. Corneal sensory impairment remains clinically important, particularly when the ophthalmic division is treated, because it may predispose patients to keratitis and corneal injury. These mixed-age estimates should not be interpreted as older-adult complication rates.

Radiofrequency lesioning may be discussed as a possible option when rapid pain relief is required, and open surgery is unsuitable or declined. However, because no included study provided separately extractable outcomes for patients aged ≥65 years, its possible role in older adults is extrapolated from mixed-age evidence and general procedural characteristics. Age-specific effectiveness and safety therefore remain uncertain, and the potential sensory consequences require individualized consideration.

#### 3.4.2. Percutaneous Balloon Compression

PBC mechanically compresses the trigeminal ganglion using a balloon catheter introduced through the foramen ovale (Figure 2c). The procedure generally provides immediate pain relief and is associated with a relatively short recovery period.

In a study of 105 patients older than 70 years, Li et al. reported an immediate pain-relief rate of approximately 97% [27]. This immediate response estimate should not be compared directly with the MVD medication-free pain-relief estimate because the outcome definitions and assessment times differed. Although the initial response is frequently favorable, recurrent pain may develop during longer follow-up. A systematic review and meta-analysis comparing PBC with radiofrequency thermocoagulation found that both procedures provided favorable short-term outcomes. PBC may be associated with lower pain scores and reduced recurrence during longer follow-up, although heterogeneity among the included studies limits definitive conclusions [25].

Facial numbness is common after PBC. Other adverse effects include dysesthesia, temporary masticatory weakness, and, rarely, injury to adjacent neural or vascular structures. Balloon inflation may provoke the trigeminocardiac reflex, resulting in bradycardia and hemodynamic instability. This risk is particularly relevant in older patients with cardiovascular disease. A randomized study found that trigeminal ganglion blockade reduced the trigeminocardiac response in older patients undergoing PBC [33].

PBC may be especially useful for patients who require rapid pain relief but have limited physiological tolerance for posterior fossa surgery. Its short procedural and recovery times must be balanced against facial sensory disturbance, cardiovascular effects during balloon inflation, and the possibility of recurrent pain.

#### 3.4.3. Percutaneous Retrogasserian Glycerol Rhizotomy

PRGR involves the injection of anhydrous glycerol into the trigeminal cistern to produce selective chemical injury to trigeminal sensory fibers. It is minimally invasive and generally requires fewer technical resources than MVD or radiosurgery [26].

No included publication provided separately extractable PRGR outcomes for patients aged ≥65 years. The available PRGR findings were therefore classified as indirect contextual evidence and should not be interpreted as estimates of effectiveness, durability, or safety specifically in older adults. In general adult populations, outcomes varied according to glycerol volume, patient positioning, TN phenotype, outcome definition, and follow-up duration. One mixed-age study suggested that PRGR may be less effective for pain predominantly involving the mandibular division [34]. In another mixed-age comparative study, the median duration of pain relief was approximately 21 months after PRGR and 20 months after PBC [28]. A further mixed-age comparison reported broadly similar pain outcomes after PRGR and PBC but differences in their complication profiles [35]. These findings are presented solely as indirect procedural context.

No included study provided age-stratified PRGR complication rates for patients aged ≥65 years. In one mixed-age cohort, hypesthesia occurred in 15%, dry eye in 5%, and meningitis in 2% of patients [34]. The duration of these complications was not consistently reported. Other reported complications included dysesthesia, local hematoma, trigeminal motor weakness, and anesthesia dolorosa, but sufficiently consistent numerical estimates were unavailable from the included sources. These figures therefore provide indirect procedural context rather than older-adult risk estimates.

PRGR may be discussed as a possible option when MVD is unsuitable and relevant institutional expertise is available. However, because no included study provided separately extractable outcomes for patients aged ≥65 years, its possible role in older adults is extrapolated from mixed-age evidence and general procedural characteristics. Age-specific effectiveness, durability, and safety remain uncertain.

### 3.5. Stereotactic Radiosurgery

SRS delivers focused ionizing radiation to a short segment of the trigeminal nerve, commonly near the root entry zone (Figure 2d). Gamma Knife and CyberKnife are the principal platforms. SRS does not require a surgical incision or general anesthesia and is usually performed as an outpatient procedure. These procedural characteristics may be relevant when operative or anesthetic burden is a concern, but they do not establish suitability, effectiveness, or safety specifically in older adults.

No included publication provided separately extractable SRS outcomes for a population defined as aged ≥65 years. The available SRS findings were therefore classified as indirect contextual evidence and should not be interpreted as estimates of effectiveness, durability, or safety specifically in older adults. Accordingly, any proposed clinical role for SRS in older adults, including its consideration when avoidance of general anesthesia or invasive surgery is important, represents extrapolation from mixed-age evidence and general procedural characteristics rather than demonstrated age-specific effectiveness or safety.

Unlike MVD and percutaneous procedures, SRS does not usually provide immediate pain relief. Clinical improvement generally develops over several weeks or months. Mixed-age studies have reported meaningful pain relief in approximately 70–80% of patients within six months; however, this estimate represents indirect contextual evidence and should not be interpreted as an older-adult treatment effect [29].

Mixed-age evidence suggests that target location may influence both pain-control durability and sensory complications. In the study by Xu et al., all patients received a maximum dose of 80 Gy; proximal targeting was associated with more durable pain relief but a higher incidence of mild-to-moderate facial numbness than distal targeting [30].

Quantitative SRS complication estimates were derived from mixed-age populations. In the 497-patient Gamma Knife cohort reported by Régis et al., the actuarial incidence of hypesthesia was 20.4% at five years and 21.1% at seven years; very bothersome facial hypesthesia occurred in 3/497 patients (0.6%) [29]. In the 99-patient study by Xu et al., mild-to-moderate facial numbness developed in 35/99 patients (35.4%), including 19/36 (52.8%) treated using a proximal target and 16/63 (25.4%) treated using a distal target [30]. In the CyberKnife cohort reported by Karam et al., new somewhat bothersome facial numbness occurred in 2/25 patients (8%) at a median follow-up of 28 months; no new very bothersome numbness or reduced corneal sensation was reported [36]. These longer-term mixed-age estimates do not establish complication rates specifically in older adults.

In mixed-age populations, CyberKnife has produced pain outcomes broadly comparable to those reported after Gamma Knife treatment, although direct comparative evidence remains limited [36]. Platform selection is frequently influenced by institutional availability, physician expertise, cost, and insurance coverage rather than established differences in clinical effectiveness.

### 3.6. Comparative Effectiveness and Treatment Selection

The included MVD–SRS comparative studies involved mixed-age, nonrandomized populations and did not provide separately extractable outcomes for a predefined older population. Their findings were therefore classified as indirect contextual evidence. In one prospective mixed-age cohort, the observed pain-free proportions after MVD were 88% at two years and 80% at five years, compared with 50% and 35%, respectively, after SRS [31]. Other mixed-age observational comparisons also reported higher pain-free rates after MVD, whereas SRS avoided the risks associated with craniotomy and general anesthesia [37,38,39].

These observed differences should not be interpreted as demonstrating causal superiority or age-specific comparative effectiveness. Treatment allocation was not randomized, and patients selected for SRS or percutaneous procedures were often older, frailer, or more medically complex than those selected for MVD. Differences in comorbidity, operative risk, TN phenotype, previous treatment, and other baseline characteristics may therefore have contributed to the reported outcomes. The available evidence does not adequately control for these sources of confounding.

Percutaneous procedures generally provide more rapid relief than SRS and impose a lower operative burden than MVD. They are particularly relevant when severe pain prevents adequate nutrition or hydration and an immediate response is required. Their principal trade-off is intentional trigeminal injury, which can cause facial numbness, corneal sensory impairment, dysesthesia, or, rarely, anesthesia dolorosa.

In applying this framework, chronological age, comorbidity burden, anesthetic risk, functional status, and formally measured frailty should be considered separately. Because validated frailty measures were rarely reported in the available studies, the influence of frailty on comparative procedural outcomes remains uncertain.

The direct older-adult evidence principally concerned MVD and PBC-related outcomes. Evidence concerning radiofrequency lesioning, PRGR, SRS, and several between-procedure comparisons was derived predominantly from mixed-age populations and was classified as indirect contextual evidence. Accordingly, the following framework combines limited direct older-adult evidence with general procedural characteristics and indirect comparative evidence. It should support individualized clinical judgment rather than be interpreted as a validated age-specific treatment algorithm.

Table 2 presents a proposed, non-graded expert-informed framework for discussing procedure selection; it is not intended as a clinical guideline or validated treatment algorithm.

Table 2 is intended only to structure individualized multidisciplinary discussion. Its statements are non-graded considerations rather than clinical recommendations. No single characteristic determines the optimal intervention, and treatment selection should integrate TN subtype, neuroimaging findings, frailty, anesthetic risk, antithrombotic status and tolerance of interruption, pain urgency, baseline facial sensation, previous interventions, anticipated life expectancy, and patient preferences. Several of these considerations are based primarily on observational or indirect evidence and remain susceptible to confounding and treatment-selection bias.

In carefully selected, medically fit older adults with classical TN and convincing neurovascular compression, direct observational evidence suggests that MVD may provide durable pain relief. Possible roles for percutaneous procedures or SRS are supported predominantly by general procedural characteristics and indirect mixed-age evidence. They should therefore be presented as cautious, individualized considerations rather than established age-specific treatment recommendations.

## 4. Discussion

The evidence base was unevenly distributed across procedures. Direct older-adult evidence concerned mainly MVD and PBC, whereas evidence concerning radiofrequency lesioning, PRGR, SRS, and most between-procedure comparisons was indirect. The clinical implications are interpreted below, with the principal limitations consolidated in Section 4.3.

### 4.1. Interpretation of the Findings

Observational evidence suggests that MVD may provide durable pain relief in carefully selected, medically fit older adults with classical TN and convincing neurovascular compression. Its principal mechanistic advantage is that it addresses neurovascular compression without intentionally injuring the trigeminal nerve. However, the available evidence does not establish comparative superiority over percutaneous procedures or SRS in older adults [18,19,20,21,22].

Direct older-adult MVD studies used different age thresholds, outcome definitions, and follow-up periods but generally reported favorable outcomes in selected patients [18,19,20,21]. Direct PBC evidence supported a high initial response and provided information about the trigeminocardiac reflex, but did not establish comparative long-term durability [27,33].

Nevertheless, neurovascular contact on MRI should not automatically be interpreted as a surgically relevant abnormality. Vascular contact may occur in asymptomatic individuals, whereas classical TN is more strongly associated with compression accompanied by morphological nerve changes. Clinical phenotype and high-resolution imaging should therefore be considered together when evaluating suitability for MVD.

The risks of MVD remain clinically important because it requires general anesthesia and posterior fossa surgery. Chronological age, comorbidity burden, anesthetic risk, functional status, and frailty should be considered separately during assessment. However, the included studies did not establish the independent predictive value of these factors, particularly formally measured frailty.

Percutaneous procedures occupy an important position between open surgery and non-invasive radiosurgery. Radiofrequency lesioning, PBC, and PRGR can provide rapid pain relief with shorter recovery periods than MVD [17,23,24,25,26,27,28,32,33,34,35]. This rapid onset is particularly valuable when pain prevents eating, drinking, medication administration, or essential oral care. In such circumstances, waiting several weeks or months for a response to SRS may be clinically inappropriate.

The benefits of percutaneous procedures must be balanced against intentional trigeminal injury. Facial numbness is common, while dysesthesia, corneal sensory impairment, masticatory weakness, and anesthesia dolorosa may occur less frequently [17,23,24,25,26,27,28,32,33,34,35]. These consequences are especially relevant in patients with pre-existing facial sensory loss or ophthalmic-division involvement. PBC also carries a risk of trigeminocardiac reflex, which requires careful anesthetic preparation in older adults with cardiovascular disease [33]. Thus, the designation “minimally invasive” should not be interpreted as meaning risk-free.

No percutaneous technique can be regarded as universally superior. Radiofrequency lesioning permits greater anatomical selectivity but may carry a higher risk of division-specific sensory injury. PBC provides rapid relief and is technically reproducible but may produce facial numbness, masticatory weakness, and significant intraoperative bradycardia. PRGR is resource-efficient and minimally invasive, but its outcomes may be less predictable, and the available age-specific evidence is limited [23,24,25,26,27,28,32,33,34,35]. The choice among these procedures should therefore depend on the affected trigeminal divisions, baseline sensory function, cardiovascular status, previous treatment, local expertise, and patient preferences.

SRS avoids incision and general anesthesia. These procedural characteristics may be relevant for patients in whom avoidance of general anesthesia or invasive surgery is clinically important, but they do not establish age-specific suitability, effectiveness, or safety. Its principal disadvantages are delayed pain relief and declining effectiveness over time [29,30,31,36,37,38,39]. The delayed therapeutic effect may be clinically important when pain interferes with nutrition, hydration, or medication use.

Several clinically important subgroups were not adequately represented in the direct older-adult evidence. None of the included studies provided separately extractable age-specific outcomes for patients with multiple-sclerosis-associated TN, continuous background pain, venous compression, recurrent TN after a previous procedure, or pre-existing facial or corneal sensory deficits. Mixed-age evidence suggests that continuous background pain and venous rather than arterial compression may be associated with less predictable outcomes after MVD, but definitions and findings were inconsistent, and their relevance to older adults remains uncertain [16,22]. In multiple-sclerosis-associated TN, pain may reflect central demyelination, neurovascular compression, or both; consequently, the relative roles of MVD, percutaneous procedures, and SRS cannot be determined from the available older-adult evidence. For recurrent TN, treatment selection may reasonably consider the previous procedure, duration of benefit, current imaging findings, operative risk, and existing sensory dysfunction, but no included older-adult study directly compared salvage strategies. Similarly, pre-existing facial numbness or corneal sensory impairment may increase concern about additional sensory injury from an ablative procedure, particularly when the ophthalmic division is involved; however, this consideration is based on procedural reasoning and mixed-age evidence rather than demonstrated age-specific comparative outcomes [17,23,24,25,26,27,28,29,30,31,32,33,34,35,36,37,38,39]. These subgroups therefore require individualized assessment and should not be incorporated into a general older-adult treatment algorithm.

### 4.2. Implications for Clinical Practice

The findings support a patient-centered and mechanism-based treatment strategy. For a medically fit older adult with classical TN, convincing neurovascular compression, and reasonable life expectancy, MVD may be discussed as a potentially definitive treatment. This consideration is supported by direct older-adult observational evidence, although its applicability to frail or medically high-risk patients remains uncertain because the reported cohorts consisted of selected surgical candidates [18,19,20,21].

In clinical practice, substantial comorbidity, limited physiological reserve, elevated anesthetic risk, or a preference to avoid craniotomy may prompt discussion of a percutaneous procedure or SRS. However, for radiofrequency lesioning, PRGR, and SRS, these considerations are extrapolated from mixed-age evidence and procedural characteristics because separately extractable outcomes for adults aged ≥65 years were unavailable. They should not be interpreted as demonstrating age-specific suitability, effectiveness, or safety.

Shared decision-making should include a clear explanation of the differences between complete medication-free pain freedom, partial pain improvement, and pain control requiring continued medication. Patients should also understand the probability of recurrence, the potential need for repeat treatment, and the risk of facial sensory disturbance. Presenting only initial response rates may overestimate the long-term benefit of percutaneous procedures and SRS.

Multidisciplinary assessment may improve treatment selection in medically complex older adults. Collaboration among neurosurgeons, neurologists, pain specialists, neuroradiologists, anesthesiologists, geriatricians, and primary care physicians can help confirm the diagnosis, evaluate frailty, optimize comorbidities, and align treatment with the patient’s functional goals.

### 4.3. Limitations

#### 4.3.1. Limitations of the Available Literature

The available literature was dominated by retrospective, single-center studies with risks of selection bias, incomplete or variable follow-up, and inconsistent reporting. Direct older-adult evidence was limited mainly to MVD and PBC, whereas evidence concerning radiofrequency lesioning, PRGR, SRS, and most between-procedure comparisons was derived from mixed-age populations and could not establish age-specific effectiveness or safety.

Comparative interpretation was further limited by confounding by indication and treatment-selection bias. Medically fit older adults were more likely to undergo MVD, whereas patients with greater comorbidity or operative risk were more often selected for percutaneous procedures or SRS. Age thresholds, definitions of medication refractoriness and treatment success, assessment times, and follow-up durations also differed substantially. Sensory complications were inconsistently defined, graded, and distinguished as transient or persistent. This clinical and methodological heterogeneity precluded quantitative pooling and requires numerical outcomes to be interpreted according to each study’s population, procedure, outcome definition, and follow-up.

Frailty, comorbidity, functional status, anesthetic risk, and chronological age were not consistently reported and should not be treated as interchangeable. No included study used a validated frailty instrument and demonstrated its association with treatment selection or procedural outcomes. Together, these limitations resulted in low or very low GRADE certainty for most procedure-specific and comparative conclusions (Supplementary Table S3).

#### 4.3.2. Limitations of the Current Review

This review was conducted as a structured narrative review rather than a formal systematic review, and its protocol was not prospectively registered. The search was restricted to PubMed/MEDLINE and English-language publications; consequently, relevant studies indexed exclusively in other databases or published in other languages may have been missed. Reference-list screening could not fully compensate for these restrictions. Although title-and-abstract screening and further eligibility assessment were performed independently by two reviewers, data were extracted by one reviewer and checked by the second rather than extracted independently in duplicate. Detailed record-level reasons for exclusion during later eligibility assessment were not prospectively documented and could not be reconstructed reliably, reducing the reproducibility and auditability of study selection. In addition, selecting and interpreting indirect contextual evidence required reviewer judgment and may have introduced residual selection or interpretive bias.

### 4.4. Future Research

Future studies should adopt standardized definitions of older age, medication-refractory TN, treatment success, and recurrence. Outcomes should preferably be reported using validated measures such as the Barrow Neurological Institute pain intensity scale and should distinguish medication-free pain freedom from pain control requiring medication.

Prospective multicenter registries would facilitate more reliable comparisons among MVD, percutaneous procedures, and SRS. These registries should record TN subtype, neurovascular compression characteristics, frailty, comorbidities, cognitive status, functional independence, baseline facial sensation, previous interventions, technical procedural details, and long-term quality of life.

Frailty should be evaluated using validated instruments rather than inferred from chronological age. Future research should also determine how imaging characteristics, including arterial versus venous compression and the presence of morphological nerve changes, interact with age and frailty to predict treatment response. Future comparative studies should incorporate patient-reported outcomes, treatment burden, time to meaningful pain relief, medication discontinuation, cost-effectiveness, and the consequences of repeat procedures.

## 5. Conclusions

Direct older-adult evidence was limited mainly to MVD and PBC, whereas evidence concerning radiofrequency lesioning, PRGR, SRS, and most between-procedure comparisons was derived largely from mixed-age populations. Observational evidence suggests that MVD may provide durable pain relief in carefully selected, medically fit older adults with classical TN and convincing neurovascular compression. Direct older-adult PBC evidence indicates rapid initial pain relief, although comparative long-term durability remains uncertain.

Percutaneous procedures can provide rapid relief but involve intentional trigeminal injury, whereas SRS avoids open surgery and general anesthesia but usually has a delayed therapeutic effect. For radiofrequency lesioning, PRGR, and SRS, these considerations reflect indirect mixed-age evidence and procedural characteristics rather than adequately established older-adult-specific effectiveness or safety. Treatment should therefore be individualized according to TN subtype, physiological reserve, comorbidities, anesthetic risk, imaging findings, pain urgency, baseline facial sensation, previous interventions, and patient preferences. Prospective age-specific comparative studies using standardized outcomes and validated frailty measures are needed.

## Figures and Tables

**Table 1:** Source-specific evidence concerning neurosurgical procedures for refractory trigeminal neuralgia relevant to older adults. (**A**) Direct older-adult evidence. (**B**) Indirect mixed-age contextual evidence.

Procedure/Source	Study Design	Population and Age Threshold	Source-Specific Finding	Follow-Up	Study-Level Appraisal	Relevant Body-Level GRADE Certainty
**(A)**						
MVD—Ferroli et al. [18]	Comparative observational study	117 patients aged >65 years; direct evidence	Complete pain relief without medication: 84.5%	7–138 months	ROBINS-I: Serious	Very low—MVD pain relief and durability
MVD—Menna et al. [19]	Retrospective comparative study	25 patients aged ≥65 years; direct evidence	Immediate pain freedom: 25/25; recurrence: 4/25 (16%)	Mean 15.60 ± 12.06 months	ROBINS-I: Serious	Very low—MVD pain relief and durability
MVD—Sekula et al. [20]	Prospective cohort plus systematic review/meta-analysis	Prospective cohort: 36 older patients; mean age 73.0 ± 5.9 years. Review eligibility: >60 years; direct evidence under the source-defined older population	Author-defined “excellent outcome”: 31/36 (86.1%)	Mean 20.0 ± 7.0 months for the older cohort	ROBINS-I and AMSTAR 2: Moderate concerns	Very low—MVD pain relief and durability
MVD—Sun et al. [21]	Systematic review and meta-analysis	681 older and 1538 younger participants across 12 studies; older-age thresholds varied and were not uniformly reported; direct synthesized evidence	Pooled immediate and long-term efficacy: 92.7% and 77.5%	Varied across included studies	AMSTAR 2: Low confidence	Very low—MVD pain relief and durability
PBC—Li et al. [27]	Retrospective elderly cohort	105 patients aged >70 years; direct evidence	Immediate pain relief: approximately 97% (reported as 97.1% in the extraction table)	Immediate outcome; longer follow-up duration not reported in the accessible source record	ROBINS-I: Serious	Very low—PBC immediate pain relief and longer-term durability
**(B)**						
Radiofrequency lesioning—Yang et al. [23]	Retrospective cohort	Mixed-age population (mean 63.7 years; range 20–90 years); indirect contextual evidence	Author-defined overall success: approximately 80%	26–95 months	ROBINS-I: Serious	Very low—radiofrequency lesioning in older adults
PRGR versus PBC—Asplund et al. [28]	Comparative observational study	206 mixed-age cases (82 PBC; 124 PRGR); indirect comparative evidence	Median pain-relief duration: approximately 21 months after PRGR and 20 months after PBC	Duration of benefit reported; exact overall follow-up not reported	ROBINS-I: Serious	Very low—comparative effectiveness of percutaneous procedures in older adults
SRS—Régis et al. [29]	Historical cohort	497 previously untreated mixed-age patients with ≥1-year follow-up (median age 68.3 years; range 28.1–93.2); indirect contextual evidence	Actuarial hypesthesia: 20.4% at 5 years and 21.1% at 7 years	Median 43.8 months; actuarial estimates at 5 and 7 years	ROBINS-I: Moderate	Very low—SRS outcomes in older adults
SRS—Xu et al. [30]	Retrospective cohort	99 mixed-age patients; age not reported in the accessible source record; indirect contextual evidence	Facial numbness: 35/99 (35.4%); 19/36 (52.8%) with proximal targeting and 16/63 (25.4%) with distal targeting	Median 33 months (range 6–124 months)	ROBINS-I: Serious	Very low—SRS outcomes in older adults
MVD versus SRS—Linskey et al. [31]	Prospective nonrandomized comparative cohort	80 mixed-age patients: MVD median age 54 years (range 36–70) and GKS median age 74 years (range 48–92); indirect comparative evidence	Pain-free rates: MVD 88% and 80% versus SRS 50% and 35% at 2 and 5 years	Mean 3.4 ± 2.14 years (range 0.17–8.5 years); outcomes at 2 and 5 years	ROBINS-I: Serious	Very low—comparative effectiveness of MVD versus SRS in older adults

Note: Panel A presents studies reporting separately extractable outcomes for populations defined using the displayed age thresholds. These thresholds are not assumed to represent clinically equivalent populations. Panel B presents mixed-age evidence used only for procedural or comparative context and not as evidence of age-specific effectiveness or safety. Study-level appraisal reflects the design-appropriate assessment reported in Supplementary Table S3, whereas GRADE certainty applies to the relevant body of evidence rather than to an individual study. Pooled evidence and its constituent primary studies should not be interpreted as independent confirmation of the same finding. Outcomes should not be compared directly because populations, study designs, outcome definitions, follow-up periods, and treatment selection differed. Abbreviations: AMSTAR 2, A Measurement Tool to Assess Systematic Reviews 2; GKS, Gamma Knife surgery; MVD, microvascular decompression; PBC, percutaneous balloon compression; PRGR, percutaneous retrogasserian glycerol rhizotomy; ROBINS-I, Risk Of Bias In Non-randomized Studies—of Interventions; SRS, stereotactic radiosurgery.

**Table 2:** Factors to consider during individualized procedural discussion.

Clinical Factor	MVD: Discussion Considerations	Percutaneous Procedure: Discussion Considerations	SRS: Discussion Considerations	Evidence Basis
Classical TN with convincing neurovascular compression	Potential relevance in medically fit patients with convincing neurovascular compression; direct older-adult evidence is observational and subject to selection bias.	A non-craniotomy option that may enter discussion when MVD is unsuitable or declined; an older-adult comparative advantage has not been established.	A non-incisional option that may enter discussion when operative burden is a concern; an older-adult comparative advantage has not been established.	MVD: limited direct older-adult observational evidence. Percutaneous procedures and SRS: indirect mixed-age evidence and clinical/procedural reasoning.
Immediate pain relief required	Produces immediate decompression when performed, but discussion must account for operative and anesthetic burden.	Typically provides rapid relief; relevance to older adults is inferred from mixed-age evidence and procedural characteristics.	Pain relief is usually delayed, which may be relevant to the timing of symptom control.	Predominantly indirect mixed-age evidence and clinical/procedural reasoning; no age-specific comparative evidence.
Frailty assessed using a validated instrument (e.g., Clinical Frailty Scale)	Increasing frailty may increase operative and anesthetic concerns; no validated TN-specific frailty cutoff exists.	Overall procedural and anesthetic tolerance remains relevant; no validated TN-specific frailty cutoff exists.	Avoidance of general anesthesia may be relevant to individualized discussion, but frailty itself has not been validated as an age-specific indication for SRS; no TN-specific frailty cutoff exists.	Clinical/procedural reasoning; no validated TN-specific cutoff or direct comparative older-adult evidence.
Major cardiopulmonary comorbidity	Requires individualized perioperative assessment of operative and anesthetic risk.	Requires assessment of sedation, hemodynamic effects, and procedure-specific risks.	Avoidance of general anesthesia and invasive surgery may be relevant to individualized discussion, but does not establish age-specific superiority or suitability.	Clinical/procedural reasoning; age-specific comparative evidence is unavailable.
Antithrombotic therapy and feasibility of interruption	Assess indication, thrombotic risk, agent, and feasibility of perioperative interruption through individualized multidisciplinary planning.	Assess bleeding and thrombotic risks and whether interruption is feasible; management depends on the procedure and institutional protocol.	Assess antithrombotic status and interruption requirements according to the treatment platform and institutional protocol.	Clinical/procedural reasoning and institutional protocols; not supported by comparative older-adult evidence in this review.
Preservation of facial sensation is a priority	Trigeminal injury is not intentional, although sensory complications remain possible.	Intentional trigeminal injury and sensory change are central considerations for discussion.	Facial numbness may occur and should be discussed.	Predominantly indirect mixed-age evidence and procedural characteristics; no age-specific comparative evidence.
Inability to maintain nutrition or hydration	Potential for immediate relief must be weighed against operative and anesthetic burden.	Potential for rapid relief may be relevant when open surgery is unsuitable, but older-adult suitability is not established.	Delayed onset of relief may be clinically relevant.	Indirect mixed-age evidence and clinical/procedural reasoning; no age-specific comparative evidence.
Limited life expectancy	Discuss anticipated durability against operative burden and the patient’s goals and time horizon.	Potential for rapid relief and procedure-specific risks may be discussed in relation to goals and time horizon.	Delayed onset of relief and non-incisional delivery may be discussed in relation to goals and time horizon.	Clinical/procedural reasoning and patient preferences; no validated age-specific selection rule.
Preference to avoid craniotomy	Involves craniotomy and may not align with this preference.	Does not require craniotomy; procedure-specific sensory and anesthetic risks remain relevant.	Does not require craniotomy or general anesthesia; delayed response and sensory effects remain relevant.	Procedural characteristics and patient preference; no evidence of age-specific effectiveness or superiority.
Preference to minimize facial numbness	Trigeminal injury is not intentional, although numbness can still occur.	Sensory change is an intended mechanism and should be explicitly discussed.	Facial numbness may occur and should be explicitly discussed.	Predominantly indirect mixed-age evidence and procedural characteristics; no age-specific comparative evidence.

Table note: This expert-informed framework is intended to support individualized shared discussion and is not a validated treatment algorithm or clinical guideline. It does not establish procedural superiority or provide treatment recommendations. “Direct older-adult evidence” indicates evidence from a source-defined older cohort or age-stratified analysis; thresholds varied among studies. “Indirect mixed-age evidence” indicates that separately extractable outcomes for adults aged ≥65 years were unavailable. “Clinical/procedural reasoning” identifies considerations based primarily on procedural characteristics or clinical judgment rather than demonstrated age-specific comparative effectiveness. MVD, microvascular decompression; PRGR, percutaneous retrogasserian glycerol rhizotomy; SRS, stereotactic radiosurgery; TN, trigeminal neuralgia.

## Data Availability

No new data were created or analyzed in this study. Data sharing is not applicable to this article.

## Supplementary Materials

The following supporting information can be downloaded at: https://www.mdpi.com/article/10.3390/brainsci16101121/s1, Table S1: PubMed search strategy, execution date, and retrieval totals; Table S2: Completed study-level extraction table, including participant numbers, age or age thresholds, and follow-up duration, for the 22 publications included in the evidence synthesis [18,19,20,21,22,23,24,25,26,27,28,29,30,31,32,33,34,35,36,37,38,39]; Table S3: Design-specific methodological quality and risk-of-bias assessment and GRADE certainty-of-evidence assessment for the included evidence [18,19,20,21,22,23,24,25,26,27,28,29,30,31,32,33,34,35,36,37,38,39]; Table S4: Summary of the quantity, applicability, follow-up, and certainty of evidence for each procedure [18,19,20,21,22,23,24,25,26,27,28,29,30,31,32,33,34,35,36,37,38,39]. All references cited in the supplementary tables are included in the main manuscript reference list.

## Abbreviations

The following abbreviations are used in this manuscript:

BNI	Barrow Neurological Institute
CISS	Constructive interference in steady state
CK	CyberKnife
CT	Computed tomography
FIESTA	Fast imaging employing steady-state acquisition
GK	Gamma Knife
ICHD-3	International Classification of Headache Disorders, third edition
MRI	Magnetic resonance imaging
MVD	Microvascular decompression
PBC	Percutaneous balloon compression
PRGR	Percutaneous retrogasserian glycerol rhizotomy
RFL	Radiofrequency lesioning
SPGR	Spoiled gradient-recalled acquisition
SRS	Stereotactic radiosurgery
TN	Trigeminal neuralgia
