# Supplementary material for: Neurosurgical Management of Refractory Trigeminal Neuralgia in Older Adults: A Structured Narrative Review
Source: Brain Sci. 2026 Oct 16;16(10):1121. doi: 10.3390/brainsci16101121 (PMC13702137; doi:10.3390/brainsci16101121)
Supplement: Supplementary file 1 [file brainsci-16-01121-s001.zip › brainsci-4537037-supplementary.pdf]

Supplementary Table S1. PubMed search strategy, original execution date, subsequent verification date, and retrieval totals

Notes: The supplementary broader search omitted the age-related concept to identify studies reporting separately extractable outcomes for older subgroups and predefined sources of indirect contextual evidence. Eligible contextual sources were restricted to evidence-based clinical guidelines, systematic reviews or meta-analyses evaluating the included procedures, direct comparative studies of eligible interventions, and studies addressing a prespecified procedural outcome for which direct older-adult evidence was unavailable. The searches were originally executed on 1 July 2026. The search syntax and PubMed retrieval totals were subsequently verified on 19 September 2026. Eligibility was restricted to English-language articles published electronically or in print by 1 July 2026. The supplementary broader search was handled as a separate, targeted search stream. Deduplication by PMID showed that 1,317 of its 3,239 records overlapped with the 1,324 principal-search records, leaving 1,922 unique supplementary-search records. These records were screened separately by the two reviewers using the predefined eligibility criteria for indirect contextual evidence. Five included publications were uniquely contributed by this search: Sarsam et al. [22], Yang et al. [23], Gunduz et al. [24], Wu et al. [25], and Kodeeswaran et al. [26].

Search	Complete PubMed search syntax	Original execution date	Subsequent verification date	Records retrieved
Principal age-focused search	("Trigeminal Neuralgia"[Mesh] OR "trigeminal neuralgia"[tiab] OR "tic douloureux"[tiab]) AND ("Aged"[Mesh] OR "Aged, 80 and over"[Mesh] OR "older adult*"[tiab] OR elder*[tiab] OR aged[tiab] OR geriatric*[tiab] OR octogenarian*[tiab]) AND ("Microvascular Decompression Surgery"[Mesh] OR "microvascular decompression"[tiab] OR MVD[tiab] OR "radiofrequency rhizotomy"[tiab] OR "radiofrequency lesion*"[tiab] OR "radiofrequency thermocoagulation"[tiab] OR "percutaneous balloon compression"[tiab] OR "balloon compression"[tiab] OR "glycerol rhizotomy"[tiab] OR "glycerol rhizolysis"[tiab] OR "Radiosurgery"[Mesh] OR "stereotactic radiosurgery"[tiab] OR "Gamma Knife"[tiab] OR CyberKnife[tiab] OR neurosurgery*[tiab])	1 July 2026	19 September 2026	1,324

Search	Complete PubMed search syntax	Original execution date	Subsequent verification date	Records retrieved
Supplementary broader search (age terms omitted)	("Trigeminal Neuralgia"[Mesh] OR "trigeminal neuralgia"[tiab] OR "tic douloureux"[tiab]) AND ("Microvascular Decompression Surgery"[Mesh] OR "microvascular decompression"[tiab] OR MVD[tiab] OR "radiofrequency rhizotomy"[tiab] OR "radiofrequency lesion*"[tiab] OR "radiofrequency thermocoagulation"[tiab] OR "percutaneous balloon compression"[tiab] OR "balloon compression"[tiab] OR "glycerol rhizotomy"[tiab] OR "glycerol rhizolysis"[tiab] OR "Radiosurgery"[Mesh] OR "stereotactic radiosurgery"[tiab] OR "Gamma Knife"[tiab] OR CyberKnife[tiab] OR neurosurgery*[tiab])	1 July 2026	19 September 2026	3,239

Supplementary Table S2. Completed study-level extraction table for the 22 publications included in the evidence synthesis

Scope: Studies supporting Sections 3.2–3.5 are summarized. "Direct" denotes a predefined older cohort or age-stratified evidence; "indirect" denotes mixed-age evidence used for procedural or comparative context.

Abbreviations: BNI, Barrow Neurological Institute; GKS/GKRS, Gamma Knife surgery/radiosurgery; MVD, microvascular decompression; NR, not reported; PBC, percutaneous balloon compression; PRGR, percutaneous retrogasserian glycerol rhizotomy; RFT, radiofrequency thermocoagulation; SRS, stereotactic radiosurgery; TN, trigeminal neuralgia.

Verification note: Participant numbers, age data, and follow-up were checked against the original article text when openly available and otherwise against the indexed original-article record. If a specific item was unavailable in the source inspected, it is stated as "not reported" rather than inferred. For systematic reviews, the number of studies and participants is reported when available. The vago-glossopharyngeal neuralgia case report AJNS-13-525 was not included because it did not meet the TN eligibility criteria.

Age-threshold note: "Direct" indicates evidence from a source-defined older population or age-stratified analysis; it does not imply clinical equivalence among populations defined using different age thresholds. The source-specific threshold or reported age definition is repeated in the final column. Where no explicit threshold was available, the reported age information is shown and no threshold was inferred.

**Study**	**Design**	**Participants**	**Age / age threshold**	**Medication-refractory definition**	**Intervention**	**Follow-up duration**	**Reported outcomes**	**Main finding relevant to this review**	**Evidence for older adults (source-specific age criterion)**
Ferroli et al., 2010 [18]	Comparative observational study	476 total: 117 older; 359 younger	Older group >65 years; younger group <65 years	Drug-resistant TN; exact medication-failure criteria not stated in the extracted report	MVD	7–138 months	Complete pain relief without medication in 84.5% of the older group, assessed over 7–138 months; complications and mortality reported.	Complete pain relief without medication was reported in 84.5% of selected patients aged >65 years over 7–138 months; age alone was not considered a contraindication.	Direct evidence: >65 years

Study	Design	Participants	Age / age threshold	Medication-refractory definition	Intervention	Follow-up duration	Reported outcomes	Main finding relevant to this review	Evidence for older adults (source-specific age criterion)
Menna et al., 2022 [19]	Retrospective comparative study	76 total: 25 older; 51 younger	Older group ≥65 years; younger group <65 years; overall mean 59.6 ± 9.79 years	Medication-refractory TN; exact medication-failure criteria not stated in the extracted report	MVD	Older group mean 15.60 ± 12.06 months; overall range 3–48 months	Immediate pain freedom at hospital discharge in 25/25 older patients; recurrence in 4/25 (16%) during a mean follow-up of 15.60 ± 12.06 months; mean follow-up BNI score and complications reported. The mean BNI score was not converted into a categorical BNI I rate.	All 25 older patients were pain-free at discharge, and 4/25 (16%) had recurrence during mean follow-up of 15.60 ± 12.06 months; the mean follow-up BNI score was not interpreted as a BNI I rate.	Direct evidence: ≥65 years
Sekula et al., 2011 [20]	Prospective study and systematic review/meta-analysis	Prospective cohort: 89 (36 older; 53 younger); meta-analysis: 8 studies, 1,334 participants	Older cohort mean 73.0 ± 5.9 years; younger cohort mean 52.9 ± 8.8 years; review eligibility >60 years	Medication-refractory TN; exact medication-failure criteria not stated in the extracted report	MVD	Older cohort mean 20.0 ± 7.0 months; review follow-up varied	Author-defined "excellent outcome" in 31/36 older patients at a mean follow-up of 20.0 ± 7.0 months; pooled major complications assessed in eight studies. "Excellent outcome" was not assumed to represent BNI I or medication-free pain freedom.	The source reported an author-defined "excellent outcome" in 31/36 older patients at 20.0 ± 7.0 months; this was not interpreted as BNI I.	Direct evidence: older cohort mean 73.0 ± 5.9 years; systematic-review eligibility >60 years

Study	Design	Participants	Age / age threshold	Medication-refractory definition	Intervention	Follow-up duration	Reported outcomes	Main finding relevant to this review	Evidence for older adults (source-specific age criterion)
Sun et al., 2022 [21]	Systematic review and meta-analysis	12 studies; 2,219 participants (681 older; 1,538 younger)	Elderly definitions varied across included studies; exact thresholds not uniformly reported	Definitions varied across the included studies	MVD	Varied across included studies; exact summary duration not reported	Author-defined pooled immediate efficacy of 92.7% and long-term efficacy of 77.5% in older patients; definitions and follow-up periods varied across included studies. These outcomes were not interpreted as BNI I.	The pooled author-defined efficacy estimates were 92.7% immediately and 77.5% long term; varying definitions and follow-up precluded interpretation as BNI I.	Direct synthesized evidence: older-age thresholds varied across included studies and were not uniformly reported
Sarsam et al., 2010 [22]	Long-term observational cohort	372 MVD operations; 319 patients contactable; 266 questionnaire respondents	Age summary not reported in the accessible source record	Medication-refractory TN; exact medication-failure criteria not stated in the extracted report	MVD	Mean postoperative follow-up 7 years; outcomes also reported at 10 years	Complete pain relief without medication 71% at 10 years; recurrence and satisfaction assessed.	Recurrence was greatest during the first 2 postoperative years and declined thereafter	Indirect/contextual
Yang et al., 2022 [23]	Retrospective cohort	392 treated; outcome analysis reported for 24 previously treated and 255 control patients	Mean 63.7 years; range 20–90 years	Refractory TN after failed medication and/or prior procedures	Radiofrequency rhizotomy	26–95 months	Source-defined significant pain improvement in 21/24 (88%) previously treated and 226/255 (89%) control patients during 26–95 months of follow-up; not presented as medication-free pain freedom.	Source-defined significant pain improvement was reported during 26–95 months of follow-up; the outcome was not restricted to older adults and was not interpreted as medication-free pain freedom.	Indirect/contextual

Study	Design	Participants	Age / age threshold	Medication-refractory definition	Intervention	Follow-up duration	Reported outcomes	Main finding relevant to this review	Evidence for older adults (source-specific age criterion)
Yao et al., 2016 [32]	Cohort study	1,354 patients	All participants ≥28 years; detailed age distribution not reported in the accessible source	Idiopathic TN selected for radiofrequency thermocoagulation; exact medication-failure criteria not stated	Radiofrequency thermocoagulation	At discharge and 1, 3, 5, 7, and 9 years	Pain relief and sensory complications reported by lesion temperature (62°C, 65°C, and 68°C).	Higher lesion temperatures may increase sensory injury	Indirect/contextual

Gunduz et al., 2021 [24]	Retrospective case series	156 patients; 209 procedures	Mean 58.03 ± 14.01 years; range 27–86 years	TN treated with radiofrequency thermocoagulation; exact medication-failure criteria not stated	Radiofrequency thermocoagulation	Assessments at 1, 3, and 5 years; longest reported pain-free observation 118 months	Early BNI outcomes, long-term pain control, recurrence, and complications were reported. Early facial hypoesthesia occurred after 193/209 procedures (92.3%), comprising two mutually exclusive author-defined categories: tactile hypoesthesia after 182/209 procedures (87.1%) and facial numbness after 11/209 procedures (5.3%). Facial numbness was included within the overall hypoesthesia total rather than reported as an additional event. The source did not provide further operational definitions distinguishing these categories. Dysesthesia was reported separately after 3/209 procedures (1.4%). Corneal hypoesthesia or reflex impairment occurred after 7/209 procedures (3.3%),	Facial sensory complications varied with lesioning parameters	Indirect/contextual

Study	Design	Participants	Age / age threshold	Medication-refractory definition	Intervention	Follow-up duration	Reported outcomes	Main finding relevant to this review	Evidence for older adults (source-specific age criterion)
							masseter weakness after 5/209 (2.4%), and temporal-muscle atrophy after 1/209 (0.5%). These were early outcomes, and their persistence was not consistently reported.		
Li et al., 2021 [27]	Retrospective elderly cohort	105 patients	>70 years	Medication-refractory TN; exact medication-failure criteria not stated	PBC	Specific follow-up duration not reported in the accessible source record	Source-defined immediate pain cure in 97.1%; recurrence and postoperative complications assessed. The immediate outcome was not interpreted as long-term medication-free pain freedom.	Source-defined immediate pain cure was 97.1% in patients aged >70 years; this did not establish long-term medication-free pain freedom.	Direct evidence: >70 years
Wu et al., 2023 [25]	Systematic review and meta-analysis	16 studies; 3,326 participants	Mixed-age studies; age criteria varied across included studies	Definitions varied across the included studies	PBC vs radiofrequency thermocoagulation	Immediate, 1 month, 3 months, 12 months, and recurrence at 1 and 2 years	Source-defined short-term efficacy of RFT and PBC was similar at the reported immediate, 1-, 3-, and 12-month assessments; recurrence was assessed at 1 and 2 years, and adverse events were compared.	Source-defined short-term efficacy was similar, with recurrence assessed at 1 and 2 years; estimates were heterogeneous and not older-adult specific.	Indirect, synthesized

Study	Design	Participants	Age / age threshold	Medication-refractory definition	Intervention	Follow-up duration	Reported outcomes	Main finding relevant to this review	Evidence for older adults (source-specific age criterion)
Zhang et al., 2022 [33]	Randomized controlled study	82 patients: 41 control; 41 trigeminal-ganglion-block	Study group mean 63.17 ± 8.26 years; control mean 65.32 ± 9.03 years	Older patients selected for PBC; exact medication-failure criteria not stated	PBC with vs without trigeminal ganglion block	Intraoperative assessment only; no long-term follow-up reported	Heart rate, mean arterial pressure, trigeminocardiac reflex, and bradycardia were assessed at seven perioperative time points.	Ganglion blockade reduced the trigeminocardiac response	Direct evidence: source-defined older PBC population; mean ages 63.17 ± 8.26 and 65.32 ± 9.03 years; explicit threshold not reported
Kodeeswaran et al., 2015 [26]	Clinical case series	93 patients	Age not reported in the accessible source record	TN selected for PRGR; exact medication-failure criteria not stated	PRGR	Mean 18.8 months	Source-defined immediate pain relief 96.8%, longer-term pain relief 89.4%, and recurrence 10.4% during a mean follow-up of 18.8 months.	The source reported immediate and longer-term pain relief of 96.8% and 89.4%, respectively, during mean follow-up of 18.8 months; these were not older-adult-specific estimates.	Indirect/contextual
Koning et al., 2015 [34]	Observational study	60 consecutive patients; questionnaire follow-up in 55 (92%)	Age not reported in the accessible source record	TN selected for glycerol rhizolysis; exact medication-failure criteria not stated	PRGR	Outcomes reported at 12 and 24 months and beyond 2 years; exact overall duration not reported	Initial pain relief 92%; pain-free survival 59% at 12 months and 53% at 24 months; outcomes assessed by division.	Effect may be reduced for predominantly V3 pain	Indirect/contextual

Study	Design	Participants	Age / age threshold	Medication-refractory definition	Intervention	Follow-up duration	Reported outcomes	Main finding relevant to this review	Evidence for older adults (source-specific age criterion)
Asplund et al., 2016 [28]	Comparative study	206 analyzed cases: 82 PBC; 124 PRGR	Age not reported in the accessible article text	Primary TN selected for percutaneous treatment; exact medication-failure criteria not stated	PBC vs PRGR	Clinical assessment at 1–3 days and 3–6 months, followed by telephone follow-up; exact overall duration not reported	Source-defined initial success was 82% after PBC and 85% after PRGR; median pain-free time was 20 and 21 months, respectively. The outcome definitions were retained without assuming equivalence to BNI I.	Source-defined initial success and median pain-free time were broadly similar; this mixed-age comparison was not interpreted as an older-adult estimate or as BNI I.	Indirect/comparative
Kouzounias et al., 2010 [35]	Comparative study	45 PBC patients (66 procedures) and 101 PRGR patients (120 attempts); 5 and 9 patients, respectively, lost to follow-up	Age not reported in the accessible source record	TN selected for percutaneous treatment; exact medication-failure criteria not stated	PBC vs glycerol rhizotomy	Exact follow-up duration not reported	Source-defined immediate pain relief was 85% after PBC and 87% after PRGR; the 50% recurrence time was 21 and 16 months, respectively. Exact overall follow-up was not reported.	Source-defined immediate pain relief and recurrence timing were retained as reported; outcomes were not assumed equivalent to BNI I and were not older-adult specific.	Indirect/comparative
Régis et al., 2016 [29]	Historical cohort; n=497	737 treated; primary analysis restricted to 497 previously untreated patients with ≥1-year follow-up	Median 68.3 years; range 28.1–93.2 years	Classical TN selected for Gamma Knife SRS; exact medication-failure criteria not stated	Gamma Knife SRS	Median 43.8 months; range 12–174.4 months	Source-defined initial pain freedom 91.75%; longer-term pain freedom, recurrence, and hypesthesia were assessed during a median follow-up of 43.8 months (range 12–174.4).	Source-defined initial and longer-term pain-freedom outcomes were reported over a median 43.8 months; they were not older-adult-specific estimates.	Indirect/contextual

Study	Design	Participants	Age / age threshold	Medication-refractory definition	Intervention	Follow-up duration	Reported outcomes	Main finding relevant to this review	Evidence for older adults (source-specific age criterion)
Xu et al., 2014 [30]	Retrospective cohort	99 patients: 36 proximal-target; 63 distal-target	Age not reported in the accessible source record	TN treated with SRS; exact medication-failure criteria not stated	SRS	Median 33 months; range 6–124 months	BNI I–IIIa maintained in 89%, 81%, and 69% at 1, 2, and 4 years; numbness 35/99.	Proximal targeting was associated with more durable pain relief but a higher incidence of mild-to-moderate facial numbness than distal targeting; 35/99 patients (35.4%) developed numbness, including 19/36 (52.8%) with proximal targeting and 16/63 (25.4%) with distal targeting	Indirect/contextual
Karam et al., 2014 [36]	Retrospective cohort	25 patients	Mean 65 years; range 43–86 years	Refractory TN treated with CyberKnife SRS	CyberKnife SRS	Median 28 months; mean 38.84 months; range 12–129 months	Freedom from severe pain was 72% at a median follow-up of 28 months; pain response, recurrence, and numbness reported. This was not interpreted as complete medication-free pain freedom.	Freedom from severe pain—not complete medication-free pain freedom—was 72% at a median follow-up of 28 months.	Indirect/contextual

Study	Design	Participants	Age / age threshold	Medication-refractory definition	Intervention	Follow-up duration	Reported outcomes	Main finding relevant to this review	Evidence for older adults (source-specific age criterion)
Linskey et al., 2008 [31]	Prospective comparative cohort	80 patients: 36 MVD; 44 GKS	MVD median 54 years (range 36–70); GKS median 74 years (range 48–92)	TN selected for MVD or Gamma Knife SRS; exact medication-failure criteria not stated	MVD vs Gamma Knife SRS	Mean 3.4 ± 2.14 years; range 0.17–8.5 years	Source-defined pain-free rates were reported initially and at 2 and 5 years; satisfaction and sensory complications were assessed. Outcomes were retained without assuming equivalence to BNI I.	Source-defined pain-free proportions favored MVD at 2 and 5 years, but nonrandom allocation and differing baseline ages limited inference; outcomes were not assumed equivalent to BNI I.	Indirect/comparative
Inoue et al., 2017 [37]	Retrospective comparative study	231 patients: 179 MVD; 52 GKS	Age data not reported in the accessible source record	TN selected for MVD or Gamma Knife SRS; exact medication-failure criteria not stated	MVD vs Gamma Knife SRS	At least 1 year; exact summary duration not reported	Source-defined initial pain-free status and subsequent recurrence or medication use were compared over at least 1 year; exact summary follow-up was not reported.	Source-defined initial pain-free status and later recurrence or medication use favored MVD durability, but nonrandomized selection limited inference; no equivalence to BNI I was assumed.	Indirect/comparative
Zeng et al., 2018 [38]	Prospective comparative study	441 patients: 220 MVD; 221 GKS	Age data not reported in the accessible source record	Primary TN selected for MVD or Gamma Knife SRS; exact medication-failure criteria not stated	MVD vs Gamma Knife SRS	2 years	Source-defined complete and partial pain relief, recurrence, and complications were compared at 2 years; complete and partial relief were retained as distinct outcomes.	At 2 years, source-defined complete relief generally favored MVD; partial relief was retained separately, and neither category was reclassified as BNI I.	Indirect/comparative

Study	Design	Participants	Age / age threshold	Medication-refractory definition	Intervention	Follow-up duration	Reported outcomes	Main finding relevant to this review	Evidence for older adults (source-specific age criterion)
Li et al., 2020 [39]	Single-institution retrospective comparison	214 patients: 111 MVD; 103 SRS	MVD median 60 years; SRS median 72 years	Type 1 idiopathic TN selected for MVD or SRS; exact medication-failure criteria not stated	MVD vs SRS	Follow-up duration not reported in the accessible source record	BNI outcomes, recurrence requiring another operation, and outcomes of MVD after failed SRS were reported; follow-up duration was not reported in the accessible source record.	Source-reported BNI outcomes and reoperation for recurrence generally favored MVD durability; follow-up duration was unavailable, and no outcome category was reclassified.	Indirect/comparative

Outcome-definition note: Author-defined terms such as “success,” “efficacy,” “meaningful pain relief,” “excellent outcome,” and “favorable outcome” retain the original source definitions and assessment times when available and should not be interpreted as BNI I or complete medication-free pain freedom unless explicitly stated.

Clinical-applicability note: For radiofrequency lesioning, PRGR, and SRS, no included study provided separately extractable outcomes for patients aged ≥65 years. Any discussion of their possible roles in older adults therefore represents cautious extrapolation from mixed-age evidence and procedural characteristics and does not establish age-specific suitability, effectiveness, or safety.

Supplementary Table S3. Design-specific risk-of-bias assessment and GRADE certainty-of-evidence assessment

Assessment framework. Randomized studies were assessed using RoB 2. Nonrandomized comparative intervention studies and observational cohorts estimating intervention effects were assessed using ROBINS-I. Uncontrolled descriptive case series were assessed using the Joanna Briggs Institute (JBI) Critical Appraisal Checklist for Case Series because ROBINS-I is not specifically designed for purely descriptive studies without a comparator. Systematic reviews were assessed using AMSTAR 2. For case series, appraisal considered clear eligibility criteria; reliable and consistent measurement of the condition and outcomes; consecutive and complete inclusion of participants; adequate reporting of clinical, demographic, and outcome information; and appropriate statistical analysis. JBI findings were reported as item-level judgments and an overall appraisal and were not converted into ROBINS-I risk-of-bias categories. "Overall judgment/appraisal" uses the terminology appropriate to the applicable tool and reflects internal validity or methodological quality rather than clinical directness or certainty of the body of evidence.

Study	Design	Tool	Selection / eligibility	Confounding / participant inclusion	Outcome measurement	Missing data / reporting	Overall judgment / appraisal	Principal basis for judgment
Ferroli et al., 2010 [18]	Comparative observational	ROBINS-I	Moderate	Serious	Moderate	Moderate	Serious	Nonrandomized age-group comparison; residual confounding and selection of surgically fit patients.
Menna et al., 2022 [19]	Retrospective comparative	ROBINS-I	Moderate	Serious	Moderate	Moderate	Serious	Single-center retrospective comparison; baseline health and treatment-selection factors were incompletely controlled.
Sekula et al., 2011 [20]	Prospective cohort plus systematic review/meta-analysis	ROBINS-I and AMSTAR 2	Moderate	Moderate	Moderate	Moderate	Moderate concerns	Prospective elderly cohort strengthened ascertainment, but sample size was small; the review inherited heterogeneity and limitations of observational studies.
Sun et al., 2022 [21]	Systematic review and meta-analysis	AMSTAR 2	Moderate	Moderate	Moderate	Serious	Low confidence	Synthesis was limited by nonrandomized primary studies, variable elderly definitions, heterogeneous outcomes, and possible publication bias.
Sarsam et al., 2010 [22]	Long-term observational cohort	ROBINS-I	Moderate	Moderate	Moderate	Serious	Serious	Long follow-up was valuable, but retrospective outcome ascertainment, attrition, and lack of a concurrent comparator limited inference.
Yang et al., 2022 [23]	Retrospective cohort	ROBINS-I	Serious	Serious	Moderate	Serious	Serious	Prior-treatment groups were clinically heterogeneous; incomplete denominators and

Study	Design	Tool	Selection / eligibility	Confounding / participant inclusion	Outcome measurement	Missing data / reporting	Overall judgment / appraisal	Principal basis for judgment
								nonrandom treatment histories increased selection and confounding risks.
Yao et al., 2016 [32]	Cohort study	ROBINS-I	Moderate	Serious	Moderate	Moderate	Serious	Treatment temperature was not randomized; lesion parameters and patient characteristics could confound outcome differences.
Gunduz et al., 2021 [24]	Retrospective case series	JBI Case Series Checklist	Eligibility criteria reported	Consecutive and complete inclusion unclear	Condition and early outcomes measured consistently	Follow-up/persistence and repeated-intervention reporting incomplete	Include, with methodological limitations	Uncontrolled retrospective series; eligibility and outcome ascertainment were described, but consecutive/complete inclusion was unclear, repeated interventions could introduce dependence, and persistence of complications was incompletely reported.
Li et al., 2021 [27]	Retrospective elderly cohort	ROBINS-I	Moderate	Serious	Moderate	Moderate	Serious	Age-specific cohort was relevant, but single-center selection, no concurrent comparator, and multivariable-model limitations reduced certainty.
Wu et al., 2023 [25]	Systematic review and meta-analysis	AMSTAR 2	Moderate	Moderate	Moderate	Serious	Low confidence	Conclusions depended mainly on heterogeneous nonrandomized studies with variable techniques, outcome definitions, and follow-up.
Zhang et al., 2022 [33]	Randomized controlled study	RoB 2	Low	Low	Some concerns	Some concerns	Some concerns	Randomization supported the perioperative comparison; blinding and reporting details were limited, and the outcome scope did not address long-term procedural effectiveness.
Kodeeswaran et al., 2015 [26]	Clinical case series	JBI Case Series Checklist	Eligibility criteria incompletely reported	Consecutive and complete inclusion unclear	Condition and outcomes generally described	Clinical characteristics, follow-up, and outcome reporting incomplete	Include, with methodological limitations	Small uncontrolled series; consecutive and complete inclusion could not be established, and limited clinical-detail, follow-up, and outcome reporting reduced confidence in the descriptive findings.

Study	Design	Tool	Selection / eligibility	Confounding / participant inclusion	Outcome measurement	Missing data / reporting	Overall judgment / appraisal	Principal basis for judgment
Koning et al., 2015 [34]	Observational study	ROBINS-I	Moderate	Serious	Moderate	Moderate	Serious	Nonrandom division-specific comparison; small subgroups and clinical/technical differences could explain observed effects.
Asplund et al., 2016 [28]	Comparative observational	ROBINS-I	Moderate	Serious	Moderate	Moderate	Serious	Procedure allocation was nonrandom and susceptible to confounding by indication despite comparative follow-up.
Kouzounias et al., 2010 [35]	Comparative observational	ROBINS-I	Moderate	Serious	Moderate	Moderate	Serious	Nonrandom procedure selection and differences in baseline characteristics and technique limited causal comparison.
Régis et al., 2016 [29]	Historical cohort	ROBINS-I	Moderate	Moderate	Moderate	Moderate	Moderate	Large cohort and long follow-up strengthened precision, but historical single-center design and absence of a concurrent comparator remained limitations.
Xu et al., 2014 [30]	Retrospective cohort	ROBINS-I	Moderate	Serious	Moderate	Moderate	Serious	Target location was not randomly assigned; technical selection and baseline differences could confound efficacy and toxicity findings.
Karam et al., 2014 [36]	Retrospective cohort	ROBINS-I	Moderate	Serious	Moderate	Moderate	Serious	Single-center uncontrolled CyberKnife cohort with heterogeneous prior treatment and limited adjustment.
Linskey et al., 2008 [31]	Prospective nonrandomized comparative cohort	ROBINS-I	Moderate	Serious	Moderate	Moderate	Serious	Prospective assessment improved data collection, but marked treatment-selection differences made confounding by indication likely.
Inoue et al., 2017 [37]	Retrospective comparative	ROBINS-I	Serious	Serious	Moderate	Moderate	Serious	Retrospective MVD–SRS allocation was strongly influenced by age, health, and clinical suitability; residual confounding was substantial.

Study	Design	Tool	Selection / eligibility	Confounding / participant inclusion	Outcome measurement	Missing data / reporting	Overall judgment / appraisal	Principal basis for judgment
Zeng et al., 2018 [38]	Prospective nonrandomized comparative	ROBINS-I	Moderate	Serious	Moderate	Moderate	Serious	Prospective follow-up did not eliminate nonrandom treatment allocation or confounding by indication.
Li et al., 2020 [39]	Single-center retrospective comparison	ROBINS-I	Serious	Serious	Moderate	Moderate	Serious	MVD and SRS recipients differed in age and likely operative fitness; multivariable adjustment could not fully address treatment-selection bias.

Interpretive note. The predominance of serious overall ROBINS-I judgments reflects nonrandom treatment allocation, confounding by indication, retrospective single-center designs, and incomplete or variable follow-up. These judgments do not imply that the reported clinical findings are invalid; rather, they limit causal comparisons among procedures. The JBI appraisals of the uncontrolled case series identified limitations in participant inclusion and reporting and should not be interpreted as estimates of comparative intervention effects. The single randomized study [33] addressed a perioperative adjunct during balloon compression and therefore does not provide randomized evidence of comparative long-term effectiveness among MVD, percutaneous procedures, and SRS.

Abbreviations: AMSTAR 2, A Measurement Tool to Assess Systematic Reviews 2; JBI, Joanna Briggs Institute; MVD, microvascular decompression; RoB 2, Cochrane Risk of Bias 2 tool; ROBINS-I, Risk Of Bias In Non-randomized Studies—of Interventions; SRS, stereotactic radiosurgery.

Table note. The domain columns are harmonized for compact presentation. For JBI-assessed case series, "Selection / eligibility" summarizes eligibility criteria; "Confounding / participant inclusion" summarizes consecutive and complete inclusion; and the final column provides the design-appropriate overall appraisal. JBI judgments were not translated into ROBINS-I categories.

Part B. GRADE certainty-of-evidence assessment

Assessment framework. Certainty was evaluated narratively for the principal procedure–outcome bodies of evidence using the GRADE domains of risk of bias, inconsistency, indirectness, imprecision, and publication bias. Because quantitative pooling was not undertaken, judgments apply to the indicated body of evidence rather than to a pooled effect estimate. Observational evidence began at low certainty and was downgraded when additional concerns were present; randomized evidence began at high certainty. No upgrading was applied because the available evidence did not demonstrate a large effect free from plausible residual confounding, a dose–response gradient, or an effect strengthened by adjustment for plausible confounding.

Interpretive note. These certainty ratings concern confidence in the stated older-adult conclusion, not whether an intervention can provide benefit in individual patients. Evidence derived only from mixed-age populations was downgraded for indirectness and was not treated as establishing age-specific effectiveness or safety. Publication bias could not be formally assessed for most bodies of evidence because few studies were available; where relevant, it was considered possible but was not used as an automatic reason for downgrading.

Body of evidence / outcome	Evidence base	Risk of bias	Inconsistency	Indirectness	Imprecision	Publication bias	Certainty	Rationale
MVD: pain relief and durability in older adults	Four direct older-adult publications [18–21], predominantly observational; heterogeneous outcome definitions and follow-up	Serious	Serious	Not serious	Serious	Possible	Very low	Selected medically fit surgical cohorts, nonrandomized designs, heterogeneous definitions, and limited sample sizes reduce confidence in the magnitude and durability of benefit.
MVD: major complications and mortality in older adults	Direct older-adult cohorts [18–20]	Serious	Not serious	Not serious	Serious	Possible	Very low	Complications were uncommon, but cohorts were selected, event counts were small, and estimates were insufficiently precise for uncommon serious harms.
PBC: immediate pain relief and longer-term durability in older adults	One direct elderly cohort [27], supplemented by indirect comparative evidence [25]	Serious	Not serious	Serious	Serious	Possible	Very low	A high initial response was reported directly, but there was no age-specific controlled comparison and longer-term

Body of evidence / outcome	Evidence base	Risk of bias	Inconsistency	Indirectness	Imprecision	Publication bias	Certainty	Rationale
								comparative evidence was indirect and heterogeneous.
PBC: trigeminocardiac-reflex reduction with trigeminal ganglion block in older adults	One randomized study addressing a perioperative adjunct [33]	Some concerns	Not assessable	Not serious	Serious	Possible	Low	Randomization supports the specific perioperative finding, but the evidence comes from one study with limited precision and does not address long-term procedural effectiveness.
Radiofrequency lesioning: effectiveness, durability, and sensory complications in older adults	Mixed-age observational studies only [23,24,32]	Serious	Serious	Serious	Serious	Possible	Very low	No separately extractable outcomes for patients aged ≥65 years were available; estimates were observational, heterogeneous, and indirect.
PRGR: effectiveness, durability, and sensory	Mixed-age observational studies only [26,28,34,35]	Serious	Serious	Serious	Serious	Possible	Very low	No separately extractable outcomes for patients aged ≥65

Body of evidence / outcome	Evidence base	Risk of bias	Inconsistency	Indirectness	Imprecision	Publication bias	Certainty	Rationale
complications in older adults								years were available; procedure and outcome differences and small cohorts limit inference.
SRS: effectiveness, durability, and sensory complications in older adults	Mixed-age observational cohorts only [29,30,36]	Serious	Serious	Serious	Serious	Possible	Very low	No separately extractable outcomes for patients aged ≥65 years were available; age-specific applicability and comparative effects remain uncertain.
Comparative effectiveness of MVD versus SRS in older adults	Mixed-age nonrandomized comparative studies [31,37–39]	Serious	Serious	Serious	Serious	Possible	Very low	Confounding by indication, baseline differences in age and operative fitness, heterogeneous follow-up, and absent age-stratified estimates preclude causal age-specific comparisons.
Comparative effectiveness of	Mixed-age comparative studies	Serious	Serious	Serious	Serious	Possible	Very low	Nonrandom procedure selection,

Body of evidence / outcome	Evidence base	Risk of bias	Inconsistency	Indirectness	Imprecision	Publication bias	Certainty	Rationale
percutaneous procedures in older adults	and one systematic review/meta-analysis [25,28,35]							heterogeneous techniques and outcomes, and lack of separately extractable older-adult results limit comparative conclusions.
Comparative effectiveness of MVD versus percutaneous procedures in older adults	No direct age-specific comparative study was identified; relevant procedural evidence was derived indirectly from the MVD and percutaneous evidence bodies [18–28,32–35].	Serious	Serious	Serious	Serious	Possible	Very low	Nonrandom treatment allocation, differences in baseline health and operative fitness, heterogeneous outcomes, and the absence of age-stratified comparisons preclude conclusions regarding comparative superiority.

GRADE, Grading of Recommendations Assessment, Development and Evaluation; MVD, microvascular decompression; PBC, percutaneous balloon compression; PRGR, percutaneous retrogasserian glycerol rhizotomy; SRS, stereotactic radiosurgery.

Supplementary Table S4. Summary of the Quantity, Applicability, Follow-up, and Certainty of Evidence for Each Procedure

Evidence bodies are separated by outcome and directness. Participant totals from evidence syntheses were not added to those of constituent primary studies. "NR" indicates that a complete value was not reported in the extracted study-level data and was not estimated.

Procedure / evidence body	Included publications	No. of publications	Participants represented	Age definition / applicability	Follow-up	Principal outcome domain	Evidence directness	GRADE certainty
MVD—pain relief and durability	[18–21]	4	178 across three primary older-adult cohorts (117 + 25 + 36); meta-analysis [21] not added	Direct older-adult evidence; cohorts used >65 or ≥65 years; synthesized evidence used varying "elderly" definitions	Ferroli: 7–138 months; Menna: mean 15.6 months (range 3–48); Sekula: mean 20.0 ± 7.0 months; synthesis varied	Pain freedom, treatment efficacy, recurrence, and durability	Direct	Very low
MVD—major complications and mortality	[18–20]	3	178 across three primary older-adult cohorts	Direct older-adult evidence; >65 or ≥65 years	As above	Major perioperative complications, cranial nerve deficits, and mortality	Direct	Very low
PBC—immediate pain relief and longer-term durability	[25,27]	2	105 in the direct cohort [27]; review-level participants [25] not added	Direct cohort aged >70 years; comparative synthesis involved mixed-age populations	Immediate outcome in [27]; longer-term follow-up varied in [25]	Immediate pain relief, recurrence, and durability	Direct for initial response; indirect for longer-term comparison	Very low
PBC—trigeminocardiac-reflex reduction with ganglion block	[33]	1	NR	Older patients undergoing PBC; exact threshold NR in extracted data	Perioperative	Trigeminocardiac reflex and hemodynamic response	Direct for the perioperative adjunct question	Low
Radiofrequency lesioning / thermocoagulation	[23,24,32]	3	At least 156 patients (209 interventions) in [24]; complete total across all studies NR	Mixed-age/general adult populations; no separately extractable outcomes for adults aged ≥65 years	Early and long-term outcomes; intervals varied or were NR	Effectiveness, durability, recurrence, and sensory complications	Indirect	Very low
PRGR	[26,28,34,35]	4	NR	Mixed-age/general adult populations; no separately extractable outcomes for adults aged ≥65 years	Varied; one comparison reported median pain-relief duration of approximately 21 months after PRGR	Pain relief, durability, recurrence, and complications	Indirect	Very low

Procedure / evidence body	Included publications	No. of publications	Participants represented	Age definition / applicability	Follow-up	Principal outcome domain	Evidence directness	GRADE certainty
SRS	[29,30,36]	3	621 (497 + 99 + 25)	Mixed-age/general adult populations; no separately extractable outcomes for adults aged ≥65 years	Up to 7 years in [29]; median 28 months in [36]; [30] interval NR in extracted data	Pain relief, delayed onset, durability, recurrence, and sensory complications	Indirect	Very low
MVD versus SRS—comparative effectiveness	[31,37–39]	4	NR	Mixed-age nonrandomized comparative populations; no separately extractable older-adult outcomes	Varied; [31] reported outcomes at 2 and 5 years	Comparative pain freedom, durability, and complications	Indirect	Very low
Percutaneous procedures—comparative effectiveness	[25,28,35]	3	NR; review-level and primary-study totals were not combined	Mixed-age populations without separately extractable older-adult outcomes	Varied; [28] reported median pain-relief duration of approximately 20 months after PBC and 21 months after PRGR	Comparative pain relief, recurrence, durability, and complications	Indirect	Very low
MVD versus percutaneous procedures—comparative effectiveness	[18–28,32–35]	No direct age-specific comparative study	Not applicable	No direct age-specific comparative population was identified	Not applicable	Comparative effectiveness and safety	Indirect comparison across separate evidence bodies	Very low

Abbreviations: GRADE, Grading of Recommendations Assessment, Development and Evaluation; MVD, microvascular decompression; NR, not reported; PBC, percutaneous balloon compression; PRGR, percutaneous retrogasserian glycerol rhizotomy; SRS, stereotactic radiosurgery.

Notes: Certainty judgments correspond to the procedure–outcome evidence bodies reported in Supplementary Table S3. Direct evidence denotes studies restricted to defined older populations or providing separately extractable older-adult outcomes. Indirect evidence denotes mixed-age or general adult evidence without separately extractable outcomes for adults aged ≥65 years. Participant counts describe the populations represented in the cited evidence and are not pooled estimates. Systematic-review totals were excluded where inclusion could duplicate primary-study participants.
